# Longitudinal Changes in Diet Cause Repeatable and Largely Reversible Shifts in Gut Microbial Communities of Laboratory Mice and Are Observed across Segments of the Entire Intestinal Tract

**DOI:** 10.3390/ijms22115981

**Published:** 2021-06-01

**Authors:** Adrian Low, Melissa Soh, Sou Miyake, Vanessa Zhi Jie Aw, Jian Feng, Adeline Wong, Henning Seedorf

**Affiliations:** 1 Temasek Life Sciences Laboratory, 1 Research Link, Singapore 117604, Singapore; adrian@tll.org.sg (A.L.); melissasoh@tll.org.sg (M.S.); sou.miyake@kaust.edu.sa (S.M.); vannyvanvann@gmail.com (V.Z.J.A.); james4fong@gmail.com (J.F.); wong.ty.a@gmail.com (A.W.); 2 Department of Biological Sciences, National University of Singapore, Singapore 117604, Singapore

**Keywords:** gastrointestinal tract, western diet, standard chow, 16S rRNA gene amplicon sequencing, dietary changes, predictive metagenomic profiling, *Muribaculaceae*

## Abstract

Dietary changes are known to alter the composition of the gut microbiome. However, it is less understood how repeatable and reversible these changes are and how diet switches affect the microbiota in the various segments of the gastrointestinal tract. Here, a treatment group of conventionally raised laboratory mice is subjected to two periods of western diet (WD) interrupted by a period of standard diet (SD) of the same duration. Beta-diversity analyses show that diet-induced microbiota changes are largely reversible (*q* = 0.1501; PERMANOVA, weighted-UniFrac comparison of the treatment-SD group to the control-SD group) and repeatable (*q* = 0.032; PERMANOVA, weighted-UniFrac comparison of both WD treatments). Furthermore, we report that diet switches alter the gut microbiota composition along the length of the intestinal tract in a segment-specific manner, leading to gut segment-specific Firmicutes/Bacteroidota ratios. We identified prevalent and distinct Amplicon Sequencing Variants (ASVs), particularly in genera of the recently described *Muribaculaceae*, along the gut as well as ASVs that are differentially abundant between segments of treatment and control groups. Overall, this study provides insights into the reversibility of diet-induced microbiota changes and highlights the importance of expanding sampling efforts beyond the collections of fecal samples to characterize diet-dependent and segment-specific microbiome differences.

## 1. Introduction

Mice are popular model organisms for gut microbiome research partially due to the anatomical similarities between their gastrointestinal tracts (GIT) and that of humans [1]. Both conventionally raised mice and germ-free mice have enabled unprecedented insights into host-microbe interactions, including demonstrated causality between microorganisms and specific host phenotypes and/or metabolic markers [2,3]. However, while conventionally raised mice have been extensively studied, most studies only examined the fecal microbiota, which may not be representative of the entire GIT. Studies have shown a high similarity between fecal and colonic microbiotas [4,5] and a greater dissimilarity between the microbiota of the lower intestinal and upper intestinal tract [5]. Given the disparate functions between segments of the GIT [6], microbes may adapt differently to changing conditions in the host. Indeed, segment specific bacterial phylotypes have been identified in healthy C57BL/6 mice [5], but it is not well established how dietary changes, such as repetitive exposure to high sucrose/high-fat substrates typical of a western diet (WD), may affect the microbiota in the different segments of the GIT. This is specifically true for some of the dominant taxa, which are not well described yet or for which isolates have only recently been obtained, e.g., members of the *Muribaculaceae* (formerly also known as S24-7 family). This family has a high prevalence in the intestinal tract of rodents, where they often constitute the major component of the gut Bacteroidota [7]. Whilst their exact function in the gut is still unclear, determining the spatial distribution along the gut would provide insight into their adaptability within the murine gut. Hence, where possible, it appears to be warranted and needed to examine the microbiota of the entire GIT to improve our understanding of the complex, gut-segment-dependent, associations and interactions between microbes and their hosts.

Diet switches have been shown to strongly and rapidly affect the composition of the fecal microbiome [8]. However, reversibility and repeatability of diet-induced changes in the gut microbiota remain to be elucidated in greater detail. Previous studies suggested that the duration of the dietary treatment, and potentially the type of experimental diet, impact the reversibility of the induced change [9,10,11]. Diets such as WD can drastically alter the gut microbiota/microbiome into configurations often associated with undesirable phenotypes such as adiposity [12], increased susceptibility to diseases [10], reduced gut length and mass [13,14]. The ability to translate these findings directly to the human gut may be limited, but they may provide leads for future research when microbiota sampling along the entire human gut becomes technically more feasible. Reversing WD-induced changes to the gut microbiota composition via a second dietary switch using a low-fat plant-based diet has been shown to ameliorate some of those undesirable phenotypes [10,15,16]. However, it is often not apparent if a reversal after a second diet switch is complete and/or if a third diet change, using the same diet as in the first diet switch, would alter the microbiota into the same composition as after the first diet switch.

This study aims to understand the reversibility and reproducibility of murine gut microbiota impacted by periods of WD interceded by standard diet (SD) and to study the spatial heterogeneity along with the GIT of WD-fed mice. 

## 2. Results

### 2.1. Effects of a Western Diet on Animal Weight and Gastrointestinal Tract Length

**Figure 1:**
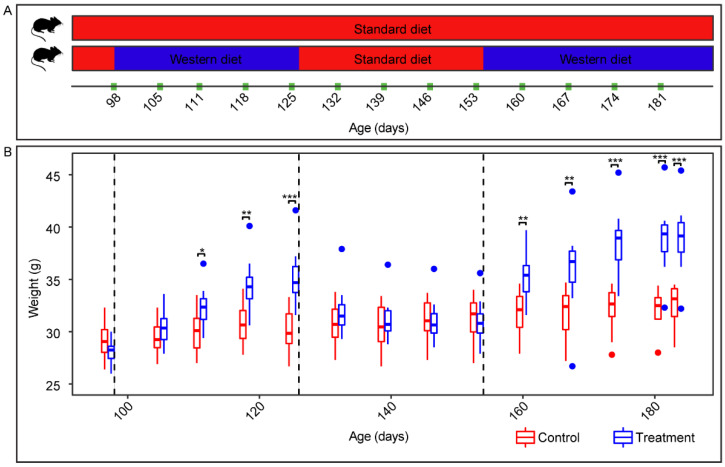
Experimental treatment and bodyweight over time. (**A**) Experimental timeline showing dietary regimens and timepoints for body weight measurements. (**B**) Boxplot depicts the weight of mice over time. Wilcoxon rank sum test was carried out to compare the weight between WD- and SD-mice (*n* = 12 mice per group). Asterisks represent significant difference where (*) denotes *q* < 0.05, (**) denotes *q* < 0.01 and (***) denotes *q* < 0.001. Dotted lines indicate a switch in diet for the treatment group.

All mice were weighed weekly to monitor bodyweight differences in response to the alternating diet regimen (Figure 1). Overall, the weight of the treatment mice (*n* = 12) exposed to two WD dietary regimens and one SD regimen was significantly different from that of the control group (*n* = 12) across 15 timepoints taken at weekly intervals (*q* = 4.63 × 10^−8^, *N* = 360; Kruskal–Wallis test; Figure 1B). The linear comparison showed that at the end of each WD regimen (day 27 and 83), the treatment mice weighed significantly more than SD-fed mice (*q*-values < 0.001; Wilcoxon rank sum test). Interestingly, the feeding of SD to treatment mice between the two WD periods led to weight loss in the treatment group animals, thus that their bodyweight at the end of the SD period was indistinguishable to that of the animals in the control group. The control mice also gradually gained weight over 84 days.

To study the outcome of the dietary regimens on gut length, the postmortem length of GIT was compared between treatment and control groups (Table S1A for endpoint gut length and bodyweight). Comparison of the individual segment lengths revealed a significantly shorter GIT in the treatment group than in the control group (*q*-values < 0.05; Table S1B for Wilcoxon rank sum test). The same trend was observed after normalization of segment length to body weight (*q*-values < 0.05; Table S1C for Wilcoxon rank sum test).

### 2.2. Effects of Diet-Switches on the Fecal Microbiota 

**Figure 2:**
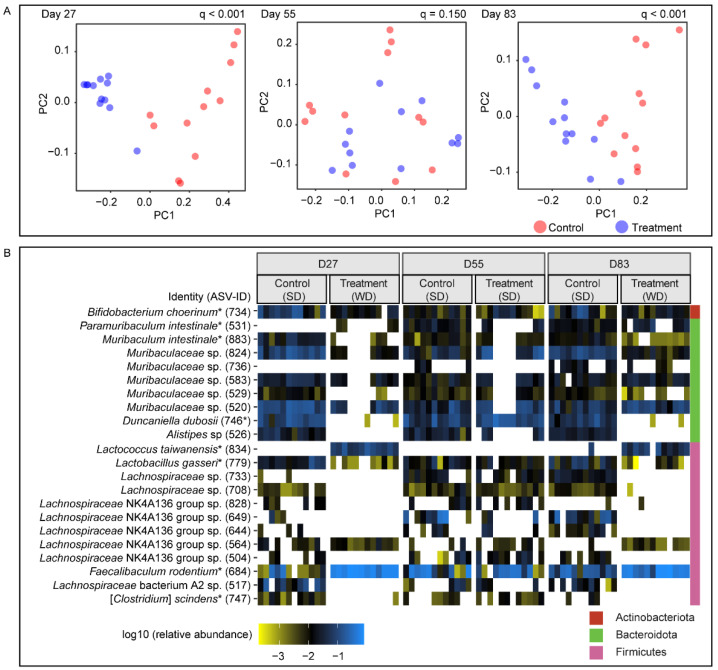
Beta-diversity between fecal microbiota of treatment (*n* = 12) and control (*n* = 12) groups. (**A**) Principal coordinate analysis (PCoA) plots for day-27, -55, and -83 based on the weighted-UniFrac distance. Statistical significance (*q*-values < 0.05) for PERMANOVA tests comparing weighted-UniFrac distance are shown. (**B**) A heatmap of 22 ASVs ≥ 0.5 relative abundance (5,203 counts per sample) per day in treatment and control. The absence of ASV is shown as white. ASVs in bold have >99% 16S rRNA gene similarity to the top BLASTn hit.

We compared weighted-UniFrac distances between treatment (WD-fed) and control groups (SD-fed) to analyze the effects of diet switches on the fecal microbiota. We were particularly interested in determining how repeatable and reversible diet-induced changes of the fecal microbiome were. At the end of both WD regimens (day 27 and 83), there was, as expected, a significant separation between treatment and control groups (*q*-values < 0.001; Table S2A for permutational multivariate analysis of variance (PERMANOVA) test; Figure 2A). However, the fecal microbiome configurations in the treatment group on days 27 and 83 were so similar that no significant difference was detectable (*q* = 0.0905; PERMANOVA), indicating high repeatability of the diet-induced change. In contrast, fecal microbiota between the same timepoints differed significantly in the control group (*q* = 0.032; PERMANOVA) (Table S2A). We also observed that the diet-induced change was highly reversible as weighted-UniFrac distances between treatment and control groups on day 55 did not significantly differ (*q* = 0.1501; PERMANOVA, Figure 2A). It is noteworthy that Bray–Curtis dissimilarity indicated a significant difference between the treatment and control group on day 55, showing that some differences between groups may persist even after four weeks (*q* = 0.0135; PERMANOVA). An ASV-level analysis of the diet-induced changes in the fecal microbiome is shown in Figure 2B (see Table S3A for mean relative abundance and taxonomic assignment of ASVs).

### 2.3. Diet-Dependent Microbiota Differences Are Observable along the Alimentary Tract

**Figure 3:**
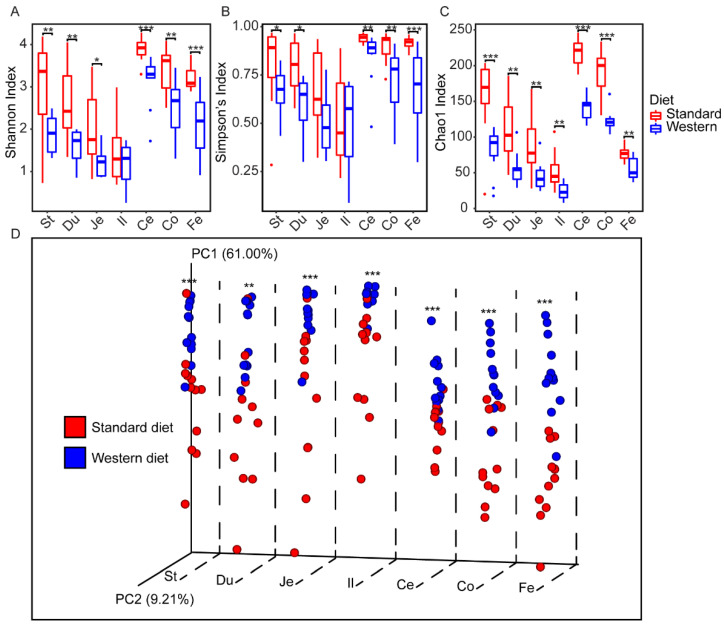
Alpha- and beta-diversity analyses of the gut segment and fecal microbiota in the western diet and standard diet-fed mice. Boxplots depict alpha-diversity shown as (**A**) Shannon, (**B**) Simpson’s, and (**C**) Chao1 indices (Table S2B,C for Wilcoxon rank sum test). (**D**) A principal coordinate analysis (PCoA) plot showing the weighted-UniFrac distance of grouped segments and fecal samples. Asterisks represent significant difference where (*) denotes *q* < 0.05, (**) denotes *q* < 0.01 and (***) denotes *q* < 0.001; PERMANOVA. St: stomach; Du: duodenum; Je: jejunum; Il: ileum; Ce: cecum; Co: colon; Fe: fecal.

We compared alpha- and beta-diversity among the GIT segments (*N* = 140) and endpoint fecal samples (*N* = 24) to identify diet-dependent and segmental differences between microbiotas. Overall, significantly reduced alpha-diversity was observed for most segments and fecal microbiotas of WD-fed mice compared to the control group according to Shannon (Figure 3A), Simpson’s (Figure 3B), and Chao1 (Figure 3C) indices. An exception to this was the ileal microbiota, where treatment and control groups shared similar species evenness (Shannon and Simpson’s indices). However, the ileum of WD-fed mice has significantly fewer rare (singletons and doubletons) ASVs than the control based on Chao1 index. 

Both Shannon and Simpson’s indexes decreased gradually from stomach to ileum with no statistically significant difference between adjacent segments (Figure 3A,B; *q*-values < 0.01; Table S2B,C for Wilcoxon rank sum tests of Shannon and Simpson’s index, respectively). A significant increase was observed from the ileum (least diverse) to the cecum (most diverse), followed by a decrease in the colon (*q*-values < 0.05; significant for Shannon index only). The Chao1 index followed a similar trend but with a greater distinction between all segments of the treatment mice (Figure 3C; Table S2D for Wilcoxon rank sum test). 

The comparison between fecal and segment microbiotas revealed different degrees of similarity in alpha diversity depending on the metric used. The treatment group fecal microbiota was highly similar to nearly all segments, except the cecum when using Simpson’s index (Table S2C). In contrast, fecal microbiota shared similar alpha diversity with fewer segments when using Shannon or Chao1 index (Shannon: stomach, duodenum, and colon, Chao1: duodenum and jejunum; Table S2B,D). The control fecal microbiota had similar alpha-diversity to the stomach (all three alpha-diversity indices), duodenum (all three alpha-diversity indices), and colon (Shannon and Simpson’s diversity). 

Beta-diversity of gut segment microbiota was analyzed using Principal Coordinate Analysis (PCoA) of weighted-UniFrac distances, which showed statistically significant separation between treatment and control groups (Figure 3D; Table S4A for PERMANOVA). Longitudinal comparison within WD-fed mice showed that adjacent segments were significantly different from one another except the jejunum vs. ileum and cecum vs. colon (Table S4B for PERMANOVA). In contrast, there was a similarity between adjacent segments of the control group except for ileum vs. cecum and cecum vs. colon (Table S4C for PERMANOVA). Fecal microbiotas were most similar to the respective colon microbiota in both groups (Table S4B,C).

### 2.4. Taxonomic Compositional Changes along the GIT and in Fecal Samples of WD-Fed Mice 

**Figure 4:**
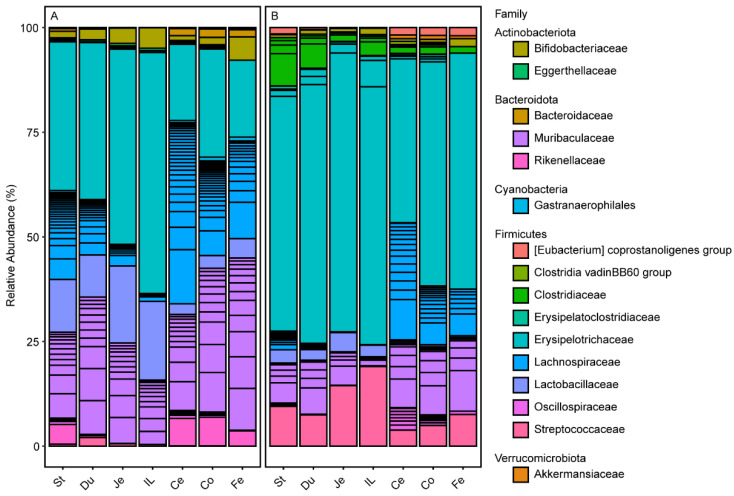
Gut microbiota classified at the family level. (**A**) Standard diet fed mice. (**B**) Western diet fed mice. Black horizontal lines on the bar-plots demarcate the different ASVs. Taxa are arranged alphabetically by phylum as indicated in legend headings. St: stomach; Du: duodenum; Je: jejunum; Il: ileum; Ce: cecum; Co: colon; Fe: fecal.

**Figure 5:**
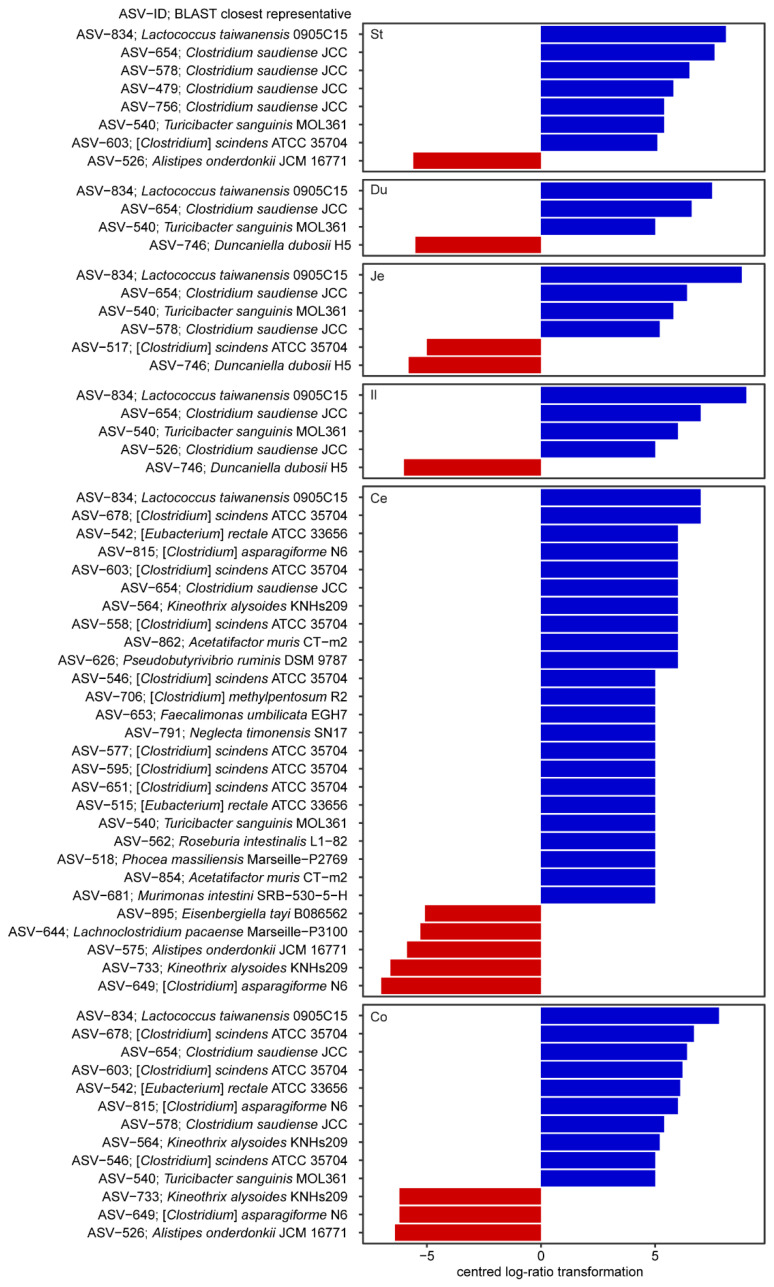
A bar-plot of differentially abundant ASVs within gut segments and fecal microbiota of mice fed a standard or western diet. Standard diet and western diet are shown as red and blue, respectively. Differential abundant ASVs were identified using analysis of the composition of microbiomes (ANCOM) method. Only statistically significant ASVs that fall within a distribution cut-off based on *W* values are shown (Table S5A for *W* values).

We analyzed microbiota at the phylum-, family- and ASV-level to determine compositional differences between WD-fed and SD-fed mice (Figure S1A, Figure 4 and Figure 5, respectively). Firmicutes and Verrucomicrobiota (replaced Verrucomicrobia in SILVA 138) showed significantly higher mean relative abundances (Firmicutes = 86.7% ± 8.8 (Std. Dev.), *n* = 82; Verrucomicrobiota = 0.39% ± 0.74) in WD-fed mice than control mice (Firmicutes = 68.3% ± 17.5, *n* = 82; Verrucomicrobiota = 0.06% ± 0.23) (*q*-values < 0.001; Table S2E). Firmicutes were prevalent in all segments, including feces in both groups. Verrucomicrobiota represented by *Akkermansiaceae* was more prevalent in the distal segments and feces compared to proximal segments of WD-fed mice. Other phyla showed significantly lower mean relative abundance than the control group (*q*-values < 0.001; Table S2E for Wilcoxon rank sum test). The exception to this was the phylum Proteobacteria, which did not differ significantly in mean relative abundances between the two groups (*q* = 0.31; Wilcoxon rank sum test). Consequently, the Firmicutes to Bacteroidota ratios (FBR) in the six gut segments as well as fecal samples revealed significantly greater variation in the treatment group than in the control group (*q* = 8.71 × 10^−5^; Kruskal–Wallis test; Figure S1B).

Greater contrast between groups and along segments can be observed at the family level. For example, Actinobacteriota represented by *Bifodobacteriaceae* and *Eggerthellaceae* showed disparate distribution within the GITs of WD- and SD-fed mice with *Eggerthellaceae* more prevalent in the stomach and duodenum of the treatment group than other segments of the treatment or control group (Figure 4).

The high prevalence of the Firmicutes in WD-fed mice can be attributed to seven families (in order of highest to lowest relative abundance): *Erysipelotrichaceae, Streptococcaceae, Clostridiaceae*, [Eubacterium]_coprostanoligenes group, *Enterococcaceae, Christensenellaceae,* and *Staphylococcaceae*. These families have significantly higher mean relative abundances in WD-fed mice than in SD-fed mice (*q*-values < 0.001; Table S2F for Wilcoxon rank sum test and mean relative abundances). In contrast, the following nine families were significantly more abundant in SD-fed mice than in WD-fed mice (in order of highest to lowest relative abundance): *Lachnospiraceae*, *Lactobacillaceae*, Clostridia_vadinBB60_group, *Butyricicoccaceae*, *Acholeplasmataceae*, *Monoglobaceae*, RF39, and *Peptococcaceae* (*q*-values < 0.01; Table S2F). Five families were similarly abundant between the groups (in order of highest to lowest relative abundance): *Oscillospiraceae, Anaerovoracaceae, Ruminococcaceae, Erysipelatoclostridiaceae,* and *Leuconostocaceae* (Table S2F). 

**Figure 6:**
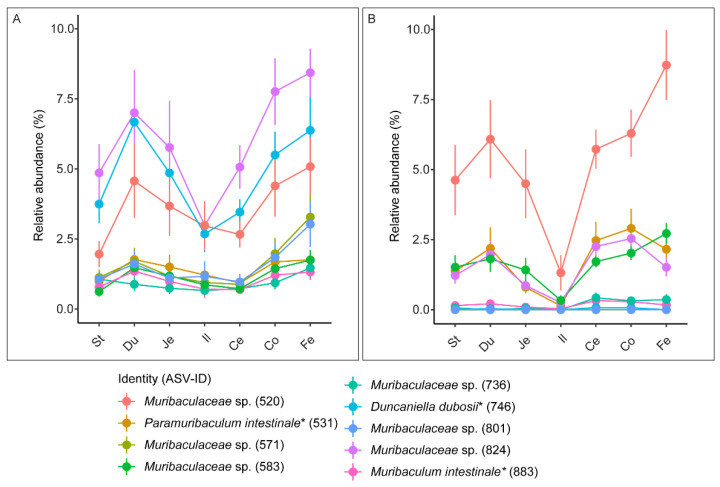
Relative abundance of nine *Muribaculaceae* ASVs along the gastrointestinal tract and feces. Shown are ASVs with ≥1% relative abundance in at least one segment/fecal group in (**A**) Control mice. (**B**) Treatment mice. Asterisks indicate ASVs with identical 16S rRNA genes to type strains. Error bars indicate standard error of the mean (*n* = 12 except duodenum: control *n* = 11; ileum: control *n* = 11, treatment *n* = 10). St: stomach; Du: duodenum; Je: jejunum; Il: ileum; Ce: cecum; Co: colon; Fe: fecal.

ASV-level analysis revealed *Erysipelotrichaceae* ASV-684 as the most abundant ASV in the treatment and control group. This ASV shares 100% identity to *Faecalibaculum rodentium* ALO17^T^ as the top BLASTn hit against the NCBI 16S rRNA gene database [17]. Moreover, analysis of the composition of microbiomes (ANCOM) identified 69 ASVs that were differentially abundant in segments of treatment and control groups (Figure 5; Table S5A). Among these was a substantial fraction of ASVs that have a low identity to cultured strains (median identity 94.2%). This includes ASVs of the family *Muribaculaceae*. We identified nine *Muribaculaceae* ASVs, which have high abundance in either a gut segment or fecal sample of the treatment or control mice (Figure 6). Three of these ASVs (ASV-531 (*Paramuribaculum intestinale* B1404^T^), ASV-746 (*Duncaniella dubosii* H5^T^), and ASV-883 (*Muribaculum intestinale* YL27^T^)) shared identical 16S rRNA genes to known isolates while the remaining ASVs were low in similarity to cultured representatives (<96%, Table S3B). All nine ASVs were detectable along the GIT of control mice, following similar relative abundance patterns for the different segments. There were fewer ASVs of similar relative abundance in treatment mice than control mice (Figure 6). Notably, ASV-801 decreased to below detection in treatment mice. ASV-746, ASV-883, and ASV-736 decreased to <1% relative abundance throughout the GIT in treatment mice.

*Muribaculaceae* ASVs of control and treatment groups reached the highest relative abundance in the duodenum of the upper intestinal tract and in the colon of the lower intestinal tract. In the treatment group, the lowest relative abundance of detectable *Muribaculaceae* ASVs was observed in the ileum, while control group *Muribaculaceae* ASVs were lowest in either ileum or cecum (Figure 6).

### 2.5. Predictive Metagenomic Profiling in Segments of Mice Fed Standard and Western Diets

The diet-induced changes in microbiota composition were also likely to affect the overall functional gene profile. PICRUSt2 was, therefore, used to predict E.C. enzymes and pathways from ASVs in gut segments [18]. The weighted mean nearest sequenced taxon index (NSTI) scores were calculated as a measure of the mean phylogenetic distances of ASVs to their closest genomic representatives, i.e., the accuracy of the prediction. The overall mean weighted NSTI was significantly lower for WD than SD fed mice (0.06 ± 0.04 (Std. Dev.); 0.10 ± 0.05, respectively; *n* = 70; *q* = 10^−5^; Wilcoxon test). The NSTI scores for segments between treatment and control group were significantly different except within ileal microbiota (*q* = 0.11; Table S2H for Wilcoxon tests; Figure S3A). A Bray–Curtis dissimilarity-based non-metric multidimensional scaling (nMDS) of predicted enzyme counts revealed clustering by similar segment types with high similarity (90%) regardless of diet (Figure S3B). We observed enzymes of E.C. class 2 transferase and E.C. 3 hydrolase that highly correlated with segments of the small intestines (rho > 0.99; Spearman; *q*-values < 0.001; Table S4D for Wilcoxon rank sum test and mean relative abundance of E.C. between small and large intestines). Conversely, we observed E.C. class 1 oxidoreductase and E.C. class 4 lyase that highly correlated with cecal and colon samples (rho >0.99; Spearman; *q*-values < 0.001; Table S4D). 

**Figure 7:**
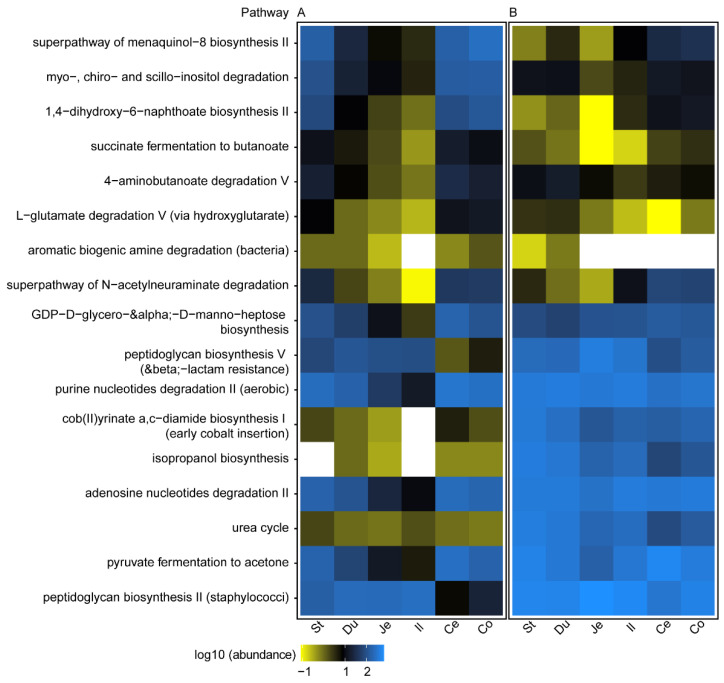
A heat-map representation of differentially abundant predicted pathways between segments of mice gastrointestinal tract of (**A**) standard or (**B**) western diet. Differential abundant MetaCyc pathways were identified using analysis of the composition of microbiomes (ANCOM) method. Only statistically significant pathways that fall within a distribution cut-off based on *W* values are shown (Table S5B for *W* values). The absence of pathway is shown as white. The mean abundance (rarefied to 132,892 counts per sample) is shown. St: stomach; Du: duodenum; Je: jejunum; Il: ileum; Ce: cecum; Co: colon.

Predicted MetaCyc pathways that were among the 20 most relatively abundant pathways in each segment revealed contrasting amino acid biosynthesis, metabolic, and nucleotide salvage pathways between WD and SD groups (Figure S3C for heatmap). Notably, a sucrose degradation III pathway was significantly enriched in WD-fed mice (*q* = 3 × 10^−5^; Wilcoxon rank sum test) from the stomach to ileum with a mean relative abundance of 0.25% ± 0.06 (Std. Dev., *n* = 46) compared to the control (0.21% ± 0.04 (Std. Dev., n = 46)) (Figure S3C). In contrast, metabolic pathways of pyruvate fermentation to acetate and lactate II (*q* = 0.009; Wilcoxon rank sum test), pyruvate fermentation to isobutanol (*q* = 0.0003; Wilcoxon rank sum test) and galactose degradation (*q* = 0.036; Wilcoxon rank sum test) were significantly lower in the treatment than control group. ANCOM analysis of predicted MetaCyc pathways between treatment and control group revealed 17 pathways unique across the segments (Figure 7; Table S5B for *W* values). ANCOM identified MetaCyc pathways that were common from stomach to ileum of treatment mice: isopropanol biosynthesis, cob(II)yrinate a,c-diamide biosynthesis I (early cobalt insertion), and urea cycle. Five pathways common between the distal segments of the treatment group were isopropanol biosynthesis, urea cycle, cob(II)yrinate a,c-diamide biosynthesis I (early cobalt insertion), peptidoglycan biosynthesis V (beta-lactam resistance) and peptidoglycan biosynthesis II (staphylococci). In the distal segments of control mice, common pathways were myo-, chiro- and scyllo-inositol degradation, superpathway of menaquinol-8 biosynthesis II, succinate fermentation to butanoate, 1,4-dihydroxy-6-naphthoate biosynthesis II and L-glutamate degradation V (via hydroxyglutarate). The pathway unique to the cecum and colon of control mice were 4-aminobutanoate degradation V and aromatic biogenic amine degradation (bacteria), respectively.

### 2.6. Discussion

In this study, we analyzed the gut microbiota of mice fed different dietary regimens to determine the effects of diet switches on the animals, the reversibility and repeatability of diet-induced microbiota changes, and the GIT segment-specific microbiota compositions in response to a diet-switch experiment. The repeated feeding of WD had profound effects on the mice in that we observed shortened gut length and altered bodyweight. The changes to bodyweight and the GIT length of laboratory mice from dietary types have been previously reported to a similar extent [13]. The increase in bodyweight was primarily attributable to an increased intake of dietary calories, and to a lesser extent to low grade inflammation leading to insulin resistance [19]. However, factors causing a reduced GIT length from WD are less well understood. A lack of fermentable fiber in WD can reduce the mass and shorten cecal and colon lengths. These phenotypes may be ameliorated by short-chain fatty acids (SCFAs) in the colon but not in cecal mass loss [20]. This indicates that different mechanisms for regulation of segment size and weight may exist and that microorganisms could contribute to some of these mechanisms, e.g., via the production of SCFAs as a byproduct of fermentation of dietary fibers [21]. The mechanisms for the length phenotypes in other GIT segments remain to be elucidated in more detail.

We show that although dietary switches induced largely reversible changes in the microbiota, a diet switch may also lead to small-scale microbiota changes that persist even after extended periods of time, i.e., four weeks. This illustrates that diet not only rapidly changes the microbiota [8] but that dietary changes may also have long-lasting effects on the microbiome composition. From the obtained data, it was not possible to infer if the microbiota would ultimately become entirely undistinguishable from the control mouse microbiota again or if the diet-switch induced changes that would persist permanently. A complete reversal of the gut microbiota may not be expected due to compositional variation among mice as they age and between cages [22,23]. Overall, our findings were consistent with previous studies indicating that WD-induced microbiota changes to the gut microbiota may take a longer time to or not fully reverse into the original state and may even be transmitted vertically to offspring [10]. It was also noteworthy that the diet composition may affect the impact and duration of the induced microbiota changes [9,14,16]. This was also evident in our study as we did not see significant microbiota differences between animals at the end of the two WD periods. The implications of these findings for diet-switch experiments in laboratory animals or for potential therapeutic interventions in humans warrant further investigations.

Lastly, we analyzed the effects of repeated feeding of WD on the composition of the microbiota and predicted microbiome in the different segments of the GIT. Regardless of the alpha-diversity index used, alpha-diversity declined from stomach to ileum, then reaching a maximum in the cecum before decreasing again in the subsequent segments and feces. This is consistent with studies of laboratory and house mice, which observed that alpha-diversity is split anatomically between small and large intestines with significantly greater species richness and evenness in the latter segments, particularly the cecum [5,24,25]. These trends are independent of diet, potentially driven by multiple factors. These factors include the anatomy and physiology of the respective GIT segment, but potentially also coprophagy, transit time, etc. However, it is also noteworthy that diet-specific effects impacted beta-diversity. WD lowered beta-diversity substantially thus that even small microbial community structure differences between adjacent segments, e.g., duodenum and jejunum, were more noticeable than in SD-fed mice.

Significant dissimilarities were also observed between fecal and stomach microbiota of WD-fed mice compared to the SD-fed control mice. This may indicate that the coprophagy behavior could be affected by the consumed diet. A reduced appetite for low fat/low sucrose diet has been observed in mice switching between WD and SD [11], which may suggest a dietary preference for WD and could provide an explanation for the observations. Ultimately, this change in eating behavior could also influence host physiology. Reduced coprophagy may not only affect the migration of fecal microbes into the gut but could limit nutrient intakes such as vitamin B12 and folic acid that are beneficial to the host wellbeing [26,27].

An increased fecal Firmicutes/Bacteroidota ratio has been reported previously and appears to be a consistent response of the murine and human fecal microbiota to WD [28]. Our study reveals that the FBR varies between gut segments. Although higher variation and mean FBR between ileal microbiota and cecal or colon microbiota have been shown in C57BL/6J mice fed a refined high fat (60%) diet [14], similar comparisons to microbiotas of the upper intestinal tract have not been demonstrated. This is important as our study shows a greater shift towards a higher FBR in most of the microbiota of the upper intestinal tract compared to the cecum and colon.

Of the Bacteroidota, the family *Muribaculaceae* have only been described recently, and few isolates have been obtained thus far [7,29,30,31,32,33]. *Muribaculaceae* were strongly impacted by WD, and this study is, to our knowledge, the first to analyze the relative abundance of *Muribaculaceae* ASVs in the different segments of the intestinal tract. Diet differentially affected the relative abundance of the different *Muribaculaceae* ASVs, e.g., ASV-520 (currently uncultured) increased significantly in relative abundance in the treatment group. Currently, it remains difficult to deduce the ecological function of specific *Muribaculaceae* ASVs in this differential response. However, the results show that diet-induced microbiota changes could increase the relative abundance of uncultured microorganisms. This approach could be used to cultivate novel members of this family, either from feces or gut segments. A comparison of the spatial distribution of different *Muribaculaceae* species in the GITs of non-mouse hosts is currently difficult due to the lack of data on this recently described family. *Muribaculaceae* have been found predominantly in rodents but less so in humans or other animals, but it appears likely that each host type harbors specific *Muribaculaceae* species [7,24,34]. 

The exact role of Firmicutes ASVs in the murine intestinal tract remains -similarly to the beforementioned Bacteroidota- often not well understood. This is also the case for ASV-684 (100% identity to *Faecalibaculum rodentium*). ASV-684 is highly prevalent throughout the alimentary tract and feces during dietary changes. *Faecalibaculum rodentium* strains have been isolated from murine feces, and the type strain has been shown to harbor genes associated with fermentation and alcohol utilization, including L-lactate and butanol dehydrogenases [35,36]. To our knowledge, the extent of *F. rodentium* prevalence throughout the alimentary tract of the laboratory murine gut has not been demonstrated before. However, it must be noted that experimental conditions, such as the supplier of mice, may lead to the observation of other phylotypes even when using C57BL/6 mice [5,37]. 

The use of PICRUSt2 to predict functional composition (E.C. numbers and MetaCyc pathways) revealed differences between proximal and distal segments. As monosaccharide absorption occurs mainly in the small intestine [38], the correlation of enzymes involved in sugar or other substrate metabolism is consistent with the functional role of the small intestine. Similarly, as distal segments are more anoxic than proximal segments, enzymes involved in redox reactions such as dehydrogenases correlate with the cecum and colon. Although a previous metagenomic study of fecal microbiomes revealed similar pathways and enzymes associated with simple sugar degradation in high fat/high sugar WD-fed mice [15], we demonstrate the spatial distribution of these enzymes within the gut. However, limitations to the accuracy of the presented predictions are given as the cultivation and characterizations of mouse microorganisms are, despite recent efforts, still not as comprehensive as databases available for human gut microbes. This is also shown by a study that compared the metagenomic profile of mice using a mouse reference database and PICRUSt2 default reference genomes, which indicated a better prediction using PICRUSt2 reference genomes [39,40]. Analytical tools for mouse gut microbiome research are likely to improve with the ongoing efforts to characterize the murine gut, including metagenomic sequencing, isolation, and mechanistic studies [30,41].

Although humans and mice share some anatomical similarities, differences in diet, behavior (e.g., coprophagy), and host-specific microbiota prevent direct inference to human health [1]. The direct translatability of our results to clinical research remains, therefore, limited. However, this study highlights the need to investigate the microbiota of other segments of the intestinal tract, potentially also humans. Particularly analysis of the microbiota in the small intestine are not performed on a routine basis. However, studies in mice have shown that the small intestine may harbor more immune system inductor sites than the colon [42]. The complex interplay of diet and microbiota in the small intestine may, therefore, be crucial for immune maturation, and a better understanding of these interactions could, therefore, also have implications for human health. 

In summary, our study shows that WD-induced microbiota changes are largely reversible and repeatable among the more abundant ASVs. Diet strongly alters relative abundances of ASVs and metabolic pathways along the lumen of the alimentary tract in a diet-dependent and in segment-specific manner. Furthermore, our study also shows that a considerable fraction of mouse gut microorganisms remain uncultured. Cultivating these microorganisms will be a prerequisite for gnotobiotic mouse experiments that aim at improving our understanding of diet-dependent and gut biogeographic microbiome differences on host physiology and immune maturation. 

## 3. Materials and Methods

### 3.1. Diet Experiment, Fecal Sampling, and Gastrointestinal Segments Harvest

A total of 24 10-week-old male C57BL/6J mice were randomly distributed into 6 cages of 4 mice each and maintained on SD for 34 days before the diet experiment commenced. Half the mice were fed ad libitum WD (carbohydrate = 49.9%, protein = 17.4%, fat = 20.0%; AIN-76A; TestDiet, St Louis, MO, USA) for 28 days, SD (carbohydrate = 53.4%, protein = 21.0%, fat = 5.0%; PicoLab Rodent Diet 20; LabDiet, St Louis, MO, USA) for another 28 days, and then WD for the last 30 days before sacrifice. The remaining mice acted as a control group and were fed SD throughout. Fresh fecal samples were collected weekly, after which the mice were weighed (Figure 1B). 

Mice were euthanized using carbon dioxide and subsequent cervical dislocation. Segments of the intestinal tract were sampled according to an established standard protocol [43]. Length of GIT segments (stomach, small intestine, cecum, and colon) were measured directly after euthanasia and dissection for individual animals. The stomach, cecum, and colon segments were grossly further subdivided into 2 equal parts, while the jejunum was divided into 3 equal parts. Individual segments or parts and fresh fecal pellets were collected into sterile 2 mL screw-cap tubes, flash-frozen in liquid nitrogen, and stored at −80 °C before DNA extraction. 

### 3.2. DNA Extraction, Indexing, and Amplicon Sequencing

Cells were lysed from sampled material using a zirconia (0.1 mm sized beads) bead-beating phenol-chloroform DNA extraction method as previously described [44]. DNA yield and purified PCR products were quantified using Quant-it Picogreen (Thermo Fisher Scientific, OR, USA). PCRs were carried out in triplicates (plus 1 no template control per sample) using primers 515F and 806R for the V4-V5 regions of the 16S rRNA gene as described in the Earth Microbiome Project protocol (Table S6A,B for fecal and segment barcodes) [45,46,47]. Each PCR contained 25 µL of 1 × Taq PCR Mastermix (Qiagen GmbH, Hilden, Germany), a final concentration of 0.2 µM of each primer, ≤20 ng of DNA, and molecular water. PCRs were carried out in 96 well plates on a Bio-Rad thermal cycler using the following conditions: 94 °C for 3 min, 35 cycles of 94 °C for 45 s, 50 °C for 60 s, 72 °C for 90 s followed by a final elongation of 72 °C for 10 min. Equimolar concentrations of PCR products were purified and pooled using a 0.8 volume of AMPure XP beads (Beckman Coulter Genomics, Danvers, MA, USA) and eluted with molecular water. Amplicons were sequenced using the 250 bp paired-end sequencing chemistry on Illumina MiSeq platforms at Axil Scientific Pte Ltd and Genome Institute of Singapore, respectively. 

### 3.3. Amplicon Sequences Processing, Microbiota and Predicted Metagenome Analyses 

Demultiplexed fastq files for the segment and fecal samples were provided by the sequencing vendor. Fastq files were processed using QIIME 2 2020.2 release with default options unless stated otherwise [48]. Primer sequences were removed from paired-end reads using the “qiime cutadapt trim-paired” command [49]. Paired-end reads were denoised, trimmed, clustered de-novo, and chimera checked using the “qiime dada2 denoise-paired” command [50] with options: “--p-trunc-len-f 180” and “--p-trunc-len-r 137” to truncate forward and reverse reads, respectively. To minimize PCR artifacts, ASVs in less than 4 samples and fewer than 12 total sequences were filtered using the “qiime feature-table filter-features” command. Paired-end reads of segment samples with multiple parts namely, stomach, jejunum, cecum, and colon were grouped by taking the mean ASV count using the “qiime feature-table group” command with the “--p-mode mean-ceiling” option (Table S6C for segment sample identities of merged sample parts). ASVs were assigned taxonomic identities using the “qiime feature-classifier classify-sklearn” command against the latest SILVA SSU for V4 region release 138 non-redundant 99% identity database, which has major changes to taxonomic nomenclature and phylogenetic lineages [51]. ASVs classified as unassigned, mitochondria, and chloroplast were removed before further analysis. After quality filtering, the segment sample mean count was 33,641 reads per sample ± 10,519 (Std. Dev., *N* = 140) with 477 ASVs of 252 bp ± 0.44 (Std. Dev.) while fecal sample mean count was 9935 reads per sample ± 3889 (Std. Dev., *N* = 72) with 384 ASVs of 207 bp ± 0.40 (Std. Dev.) (Table S6A,C for fecal and segment read count). We combined endpoint segment and fecal quality filtered feature tables and representative sequence files using “qiime feature-table merge” and “qiime feature-table merge-seqs”, respectively. The merged representative sequences were exported, aligned using the MUSCLE alignment tool and ends were trimmed to 201 bps using MEGA X version 10.2.0 [52,53]. The aligned fasta file was imported into QIIME 2 and aligned masking was performed using the “qiime alignment mask” command with the following options “--p-max-gap-frequency 0.2” to retain columns with no more than 20% gaps and “--p-min-conservation 0.8” to retain columns with at least 80% nucleotide. Unrooted and rooted phylogenetic trees for UniFrac distances were generated using “qiime phylogeny fasttree” and “qiime phylogeny midpoint-root” commands, respectively. A maximum-likelihood tree was generated using the masked aligned sequences with “qiime phylogeny raxml-rapid-bootstrap” command with the options “--p-seed 477” and “--p-rapid-bootstrap-seed 898” to construct a reproducible tree, “--p-bootstrap-replicates 1000” for 1000 bootstrapping replicates and “--p-substitution-model GTRCAT” for the GTR-CAT tree model [54]. The maximum likelihood tree was used to find identical sequences between fecal and segment dataset, and where the sequences were identical, the ASV identity from the fecal dataset was used for consistency. 

PICRUSt2 was used to predict enzymes (E.C. numbers) and MetaCyc metabolic pathways [18,55] of segment microbiota via the default pipeline using the “picrust2_pipeline.py” script. Briefly, the PICRUSt2 script ran the following commands, “place_seqs.py” aligned ASVs to reference phylogeny, “hsp.py” obtained normalized 16S rRNA gene copies based on the predicted genome to calculate NSTI values, E.C. abundances per genome. The command “pathway_pipeline.py” predicted the MetaCyc pathways from E.C. numbers, “add_descriptions.py” was used to generate enzymes and pathways output files. 

ASVs featured in heatmaps and ANCOM analyses were further annotated by taking the top BLASTn hit against the NCBI 16S rRNA gene database [17]. Phylogenetic lineages of *Muribaculaceae* and *Lactobacilli* were manually curated to identify recently recognized bacterial species or formal changes to nomenclature [7,30,31,33,56]. The “qiime emperor plot” command was used to generate custom PCoA plots of dissimilarity matrices with categorical groups on the x-axis against the first principal coordinate (PC1) on the y-axis and PC2 on the z-axis. Emperor plots were visualized at https://view.qiime2.org/ (accessed on 11 May 2021) [57]. All alpha-diversity metrices, conventional PCoA plots, heatmaps, bar graphs and boxplots were generated using R and relevant R packages, including ggplot2, phyloseq, reshape2, tidyverse, and qiime2R [58,59,60,61,62,63]. 

### 3.4. Statistical Analysis

R was used to perform statistical operations for mean, standard deviation, Kruskal–Wallis [64] and Wilcoxon rank sum tests with Benjamini–Hochberg false discovery rate (FDR) [65,66] based on counts from rarefied tables of segment and fecal (2945 counts per sample) and fecal only (5203 counts per sample). Pairwise PERMANOVA test was performed using the “qiime diversity beta-group-significance” command at 9999 permutations [67]. An FDR-corrected *p*-value (*q* < 0.05) was considered as statistically significant. A Bray–Curtis similarity matrix of square-root transformed values of a rarefied table (716,085 counts per sample) of E.C. was visualized on a nMDS plot using PRIMER-E version 6.1.16 [68]. Other PRIMER-E functions that used the Bray–Curtis similarity matrix of E.C enzymes were Spearman’s rank correlation test between enzymes and sample groups and the Similarity Profile Analysis (SIMPROF) to find significant (*p* < 0.05) clusters of samples using 999 permutations. Differential abundance tests were performed on centered-log transformed counts of ASVs and predicted MetaCyc pathways using ANCOM to compare between treatment and control groups [69]. 

## Data Availability

FastQ files were uploaded to NCBI GenBank under BioProject ID PRJNA503296 SRA accession numbers SRX4970431-SRX4970677 for segments and SAMN13908703-SAMN13908726, SAMN13908823-SAMN13908846, SAMN13908943-SAMN13908966 for fecal samples.

## Supplementary Materials

The following are available online at https://www.mdpi.com/article/10.3390/ijms22115981/s1.
