# Supplementary material for: Longitudinal Changes in Diet Cause Repeatable and Largely Reversible Shifts in Gut Microbial Communities of Laboratory Mice and Are Observed across Segments of the Entire Intestinal Tract
Source: Int J Mol Sci. 2021 Jun 1;22(11):5981. doi: 10.3390/ijms22115981 (PMC8198505; doi:10.3390/ijms22115981)
Supplement: Supplementary file 1 [file ijms-22-05981-s001.zip › Table S2.pdf]

Table S2A. PERMANOVA tests of the weighted-UniFrac distance of fecal samples.

Group 1	Group 2	Sample size	Permutations	pseudo-F	p-value	q-value
Treatment group day 27, end of 1st western diet regimen	Control group day 27	24	9999	61.204	0.0001	0.0003
Treatment group day 55, end of standard diet regimen	Control group day 55	24	9999	1.854	0.1501	0.1501
Treatment group day 83, end of 2nd western diet regimen	Control group day 83	24	9999	21.354	0.0001	0.0003
Treatment group day 27, end of 1st western diet regimen	Treatment group day 83, end of 2nd western diet regimen	24	9999	3.143	0.072	0.0905
Control group day 27	Control group day 83	24	9999	4.159	0.023	0.032

Red highlights significance FDR-adjusted *q*-value < 0.05

Table S2B. Wilcoxon rank sum test comparing Shannon index between different GIT segments and diets.

Segment, Diet	Cecum, Standard	Cecum, Western	Colon, Standard	Colon, Western	Duodenum, Standard	Duodenum, Western	Fecal, Standard	Fecal, Western	Ileum, Standard	Ileum, Western	Jejunum, Standard	Jejunum, Western	Stomach, Standard
Cecum.Western	0.00017	-	-	-	-	-	-	-	-	-	-	-	-
Colon.Standard	0.01725	0.23685	-	-	-	-	-	-	-	-	-	-	-
Colon.Western	9.60E-06	0.00838	0.00406	-	-	-	-	-	-	-	-	-	-
Duodenum.Standard	0.00579	0.18782	0.05405	0.77392	-	-	-	-	-	-	-	-	-
Duodenum.Western	6.70E-06	9.20E-05	6.70E-06	0.00326	0.00838	-	-	-	-	-	-	-	-
Fecal. Standard	0.00013	0.40012	0.21619	0.0049	0.16879	6.70E-06	-	-	-	-	-	-	-
Fecal. Western	6.70E-06	0.00091	0.00054	0.25872	0.20821	0.09488	0.00091	-	-	-	-	-	-
Ileum.Standard	9.60E-06	0.00011	8.10E-05	0.00704	0.00643	0.40012	5.00E-05	0.0846	-	-	-	-	-
Ileum.Western	1.70E-05	3.10E-05	1.70E-05	0.00029	0.00022	0.03779	1.70E-05	0.01007	0.75396	-	-	-	-
Jejunum.Standard	9.60E-06	0.0026	0.00041	0.13069	0.11981	0.46007	0.00041	0.75396	0.11981	0.02746	-	-	-
Jejunum.Western	6.70E-06	9.60E-06	6.70E-06	9.20E-05	0.00016	0.0424	6.70E-06	0.01007	0.81274	0.92287	0.02757	-	-
Stomach.Standard	0.02001	0.89725	0.64673	0.11981	0.4315	0.00157	0.81669	0.03215	0.00473	0.00054	0.02001	0.00054	-
Stomach.Western	6.70E-06	0.00017	6.70E-06	0.0373	0.05405	0.16366	6.70E-06	0.61166	0.09488	0.0125	0.75396	0.00031	0.00704

Red highlights significance FDR-adjusted *q*-value < 0.05

Table S2C. Wilcoxon rank sum test comparing Simpson index between different GIT segments and diets.

Segment, Diet	Cecum, Standard	Cecum, Western	Colon, Standard	Colon, Western	Duodenum, Standard	Duodenum, Western	Fecal, Standard	Fecal, Western	Ileum, Standard	Ileum, Western	Jejunum, Standard	Jejunum, Western	Stomach, Standard
Cecum,Western	1.25E-03	-	-	-	-	-	-	-	-	-	-	-	-
Colon,Standard	1.48E-01	2.57E-01	-	-	-	-	-	-	-	-	-	-	-
Colon,Western	1.30E-05	2.50E-02	4.07E-03	-	-	-	-	-	-	-	-	-	-
Duodenum,Standard	1.35E-02	6.44E-01	1.68E-01	2.93E-01	-	-	-	-	-	-	-	-	-
Duodenum,Western	1.10E-05	4.30E-04	1.30E-05	7.86E-02	1.91E-02	-	-	-	-	-	-	-	-
Fecal, Standard	3.97E-02	1.64E-01	9.18E-01	2.40E-04	1.38E-01	1.10E-05	-	-	-	-	-	-	-
Fecal, Western	1.20E-04	2.18E-02	1.50E-03	7.35E-01	2.71E-01	3.54E-01	1.00E-03	-	-	-	-	-	-
Ileum,Standard	1.30E-05	1.30E-03	3.00E-04	9.67E-02	1.32E-02	4.95E-01	6.70E-05	2.05E-01	-	-	-	-	-
Ileum,Western	2.20E-05	4.00E-04	2.20E-05	2.36E-02	5.51E-03	2.88E-01	2.20E-05	9.67E-02	9.13E-01	-	-	-	-
Jejunum,Standard	8.00E-05	3.97E-02	4.07E-03	6.07E-01	1.38E-01	6.35E-01	1.25E-03	9.18E-01	1.87E-01	1.93E-01	-	-	-
Jejunum,Western	1.10E-05	1.70E-04	1.30E-05	6.02E-03	8.10E-04	1.25E-01	1.10E-05	7.86E-02	9.38E-01	9.38E-01	6.30E-02	-	-
Stomach,Standard	1.48E-01	7.72E-01	7.35E-01	1.38E-01	6.13E-01	1.59E-02	8.55E-01	8.74E-02	1.91E-02	5.51E-03	7.05E-02	1.50E-03	-
Stomach,Western	1.10E-05	1.00E-03	8.00E-05	2.38E-01	6.95E-02	3.54E-01	1.10E-05	7.35E-01	2.50E-01	8.64E-02	9.77E-01	1.35E-02	3.49E-02

Red highlights significance FDR-adjusted *q*-value < 0.05

Table S2D. Wilcoxon rank sum test comparing Chao1 index between different GIT segments and diets.

Segment, Diet	Cecum, Standard	Cecum, Western	Colon, Standard	Colon, Western	Duodenum, Standard	Duodenum, Western	Fecal, Standard	Fecal, Western	Ileum, Standard	Ileum, Western	Jejunum, Standard	Jejunum, Western	Stomach, Standard
Cecum,Western	4.00E-06	-	-	-	-	-	-	-	-	-	-	-	-
Colon,Standard	5.31E-02	1.49E-03	-	-	-	-	-	-	-	-	-	-	-
Colon,Western	4.00E-06	2.66E-02	2.00E-05	-	-	-	-	-	-	-	-	-	-
Duodenum,Standard	6.10E-06	6.94E-02	4.50E-04	1.17E-01	-	-	-	-	-	-	-	-	-
Duodenum,Western	4.00E-06	4.00E-06	4.00E-06	6.10E-06	1.65E-03	-	-	-	-	-	-	-	-
Fecal, Standard	4.00E-06	4.00E-06	4.00E-06	4.00E-06	5.31E-02	1.84E-03	-	-	-	-	-	-	-
Fecal, Western	4.00E-06	4.00E-06	4.00E-06	4.00E-06	1.34E-03	9.53E-01	2.29E-03	-	-	-	-	-	-
Ileum,Standard	6.10E-06	6.10E-06	6.10E-06	1.20E-05	3.71E-03	9.77E-01	1.01E-02	5.50E-01	-	-	-	-	-
Ileum,Western	2.20E-04	2.20E-04	2.20E-04	2.20E-04	3.00E-04	1.49E-03	2.20E-04	5.30E-04	7.92E-03	-	-	-	-
Jejunum,Standard	4.00E-06	1.49E-03	4.90E-05	1.97E-02	2.79E-01	2.31E-02	7.81E-01	5.31E-02	2.88E-02	8.10E-04	-	-	-
Jejunum,Western	1.10E-04	1.10E-04	1.10E-04	1.10E-04	9.60E-04	2.35E-01	7.70E-04	7.84E-02	3.93E-01	1.41E-02	6.35E-03	-	-
Stomach,Standard	2.10E-04	3.06E-02	7.84E-02	2.82E-03	2.48E-02	4.50E-04	4.50E-04	4.50E-04	6.00E-04	8.10E-04	2.82E-03	1.10E-03	-
Stomach,Western	4.00E-06	4.00E-06	4.00E-06	4.90E-05	2.55E-01	3.78E-02	2.20E-01	1.97E-02	3.90E-02	2.24E-03	9.77E-01	1.22E-02	3.50E-04

Red highlights significance FDR-adjusted *q*-value < 0.05

Table S2E. Pairwise phylum level Wilcoxon rank sum test between treatment and control groups.

Segment, Diet	Actinobacteriota, Standard diet	Bacteroidota, Standard diet	Cyanobacteria, Standard diet	Firmicutes, Standard diet	Proteobacteria Standard diet	Verrucomicrobiota, Standard diet
Actinobacteriota, Western diet	3.40E-05	-	-	-	-	-
Bacteroidota, Western diet	-	4.60E-13	-	-	-	-
Cyanobacteria, Western diet	-	-	2.10E-11	-	-	-
Firmicutes, Western diet	-		-	1.70E-13	-	-
Proteobacteria, Western diet	-	-	-	-	0.31	-
Verrucomicrobiota, Western diet	-	-	-	-	-	7.00E-06

Red highlights significance FDR-adjusted *q*-value < 0.05

Table S2F.Family level Wilcoxon rank sum test between treatment and control groups.

	mean rel. abundance ± standard deviation WD (n = 82)	SD (n = 82)	Wilcoxon B.H correction q-value
**Firmicutes**			
Erysipelotrichaceae	55.4 ± 19.86	30.21 ± 26.15	1.00E-09
Streptococcaceae	8.96 ± 10.21	0.462 ± 2.30	<2e-16
Clostridiaceae	4.51 ± 7.02	0.018 ± 0.06	<2e-16
[Eubacterium] coprostanoligenes gr	1.03 ± 1.19	0.15 ± 0.16	8.30E-06
Enterococcaceae	0.125 ± 0.54	0	2.80E-07
Christensenellaceae	0.0765 ± 0.11	0.0003 ± 0.003	2.30E-13
Staphylococcaceae	0.037 ± 0.01	0.01 ± 0.04	0.00011
Lachnospiraceae	10.91 ± 12.92	24.78 ± 20.67	1.30E-06
Lactobacillaceae	2.23 ± 12.5	8.86 ± 4.62	8.10E-11
Clostridia UCG-014	0.042 ± 0.12	0.51 ± 0.57	<2e-16
Butyricicoccaceae	0.029 ± 0.054	0.331 ± 0.34	1.90E-14
Clostridia vadinBB60 group	0.14 ± 0.44	0.30 ± 0.41	3.10E-05
Acholeplasmataceae	0.0009 ± 0.007	0.146 ± 0.239	1.90E-14
Monoglobaceae	0	0.12 ± 0.13	<2e-16
RF39	0.005 ± 0.02	0.064 ± 0.107	2.50E-08
Peptococcaceae	0.013 ± 0.031	0.035 ± 0.059	6.60E-03
Oscillospiraceae	2.13 ± 2.66	1.42 ± 1.76	0.32
Anaerovoracaceae	0.66 ± 0.1	0.07 ± 0.08	0.28
Ruminococcaceae	0.59 ± 0.83	0.51 ± 0.51	0.24
Erysipelatoclostridiaceae	0.325 ± 0.43	0.263 ± 0.30	0.51
Leuconostocaceae	0	0.002 ± 0.012	0.083
**Bacteroidota**			
Bacteroidaceae	0	0.74 ± 1.72	1.10E-06
Rikenellaceae	0.33 ± 8.32	2.64 ± 2.62	2.90E-16
Muribaculaceae	11.33 ± 8.32	24.51 ± 14.27	3.90E-10
**Actinobacteriota**			
Bifidobacteriaceae	0.96 ± 0.80	2.63 ± 3.94	3.90E-03
Coriobacteriales Incertae Sedis	0	0.0014 ± 0.007	4.40E-02
Eggerthellaceae	0.32 ± 0.30	0.81 ± 0.93	3.80E-08
Intrasporangiaceae	0.004 ± 0.003	0.0004 ± 0.024	0.56
Microbacteriaceae	0	0.002 ± 0.0013	0.16
**Cyanobacteria**			
Gastranaerophilales	0	0.33 ± 0.59	2.10E-11
**Proteobacteria**			
Beijerinckiaceae	0.009 ± 0.047	0.0009 ± 0.008	0.31
Sphingomonadaceae	0.002 ± 0.015	0.0009 ± 0.007	1
**Verrucomicrobiota**			
Akkermansiaceae	0.393 ± 0.74	0.06 ± 0.24	7.00E-06

Red highlights significance FDR-adjusted *q*-value < 0.05
Blue indicates significantly higher mean for WD
Green indiciates signfiicantly higher mean for SD

Table S2G. Wilcoxon rank sum test between ratios of Firmicutes: Bacteroidota ASV counts.

	Stomach.Control (n=12)	Dudenum.Control (n=11)	Jejunum.Control (n=12)	Ileum.Control (n=11)	Cecum.Control (n=12)	Colon.Control (n=12)	Fecal.Control (n=12)
Stomach.Treatment (n=12)	9.48E-03	-	-	-	-	-	-
Duodenum.Treatment (n=12)	-	1.91E-02	-	-	-	-	-
Jejunum.Treatment (n=12)	-	-	2.06E-03	-	-	-	-
Ileum.Treatment (n=10)	-	-	-	1.84E-03	-	-	-
Cecum.Treatment (n=12)	-	-	-	-	4.21E-03	-	-
Colon.Treatment (n=12)	-	-	-	-	-	4.10E-04	-
Fecal.Treatment (n=12)	-	-	-	-	-	-	7.70E-05

Red highlights significance FDR-adjusted *q*-value < 0.05

Table S2H. Wilcoxon rank sum test of NSTI values between segments and diets

Segment, Diet	Stomach, Standard (n=12)	Duodenum, Standard (n=11)	Jejunum, Standard (n=12)	Ileum, Standard (n=11)	Cecum, Standard (n=12)	Colon, Standard (n=12)
Stomach,Western (n=12)	0.012	-	-	-	-	-
Duodenum,Western (n=12)	-	0.0045	-	-	-	-
Jejunum,Western (n=12)	-	-	0.0068	-	-	-
Ileum,Western (n=10)	-	-	-	0.11	-	-
Cecum,Western (n=12)	-	-		-	1.40E-05	-
Colon,Western (n=12)		-	-	-	-	3.30E-05

Red highlights significance FDR-adjusted *q*-value < 0.05
