# Supplementary material for: Longitudinal Changes in Diet Cause Repeatable and Largely Reversible Shifts in Gut Microbial Communities of Laboratory Mice and Are Observed across Segments of the Entire Intestinal Tract
Source: Int J Mol Sci. 2021 Jun 1;22(11):5981. doi: 10.3390/ijms22115981 (PMC8198505; doi:10.3390/ijms22115981)
Supplement: Supplementary file 1 [file ijms-22-05981-s001.zip › Table S4.pdf]

Table S4A. Pairwise PERMANOVA test comparing weighted-UniFrac distances between segments of western diet and standard diet.

Group 1 Western diet	Group 2 Standard diet	Sample size	Permutations	pseudo-F	p-value	q-value
Stomach	Stomach	24	9999	11.592	0.0004	0.0009
Duodenum	Duodenum	23	9999	10.843	0.0008	0.0015
Jejunum	Jejunum	24	9999	19.428	0.0001	0.0003
Ileum	Ileum	21	9999	7.624	0.0002	0.0005
Cecum	Cecum	24	9999	14.570	0.0001	0.0003
Colon	Colon	24	9999	18.502	0.0002	0.0005
Fecal	Fecal	24	9999	25.171	0.0002	0.0005

Red highlights significance FDR-adjusted *q*-value < 0.05

Table S4B. Pairwise PERMANOVA test comparing weighted-UniFrac distances between segments of western diet.

Group 1	Group 2	Sample size	Permutations	pseudo-F	p-value	q-value
Stomach	Duodenum	23	9999	12.380	0.0003	0.0007
	Jejunum	24	9999	3.863	0.0088	0.0129
	Ileum	22	9999	6.731	0.0006	0.0012
	Cecum	24	9999	15.685	0.0002	0.0005
	Colon	24	9999	5.5668	0.0086	0.0129
	Fecal	24	9999	3.6474	0.0372	0.0451
Duodenum	Jejunum	24	9999	3.220	0.0353	0.0434
	Ileum	22	9999	7.472	0.0016	0.0030
	Cecum	24	9999	18.079	0.0002	0.0005
	Colon	23	9999	6.088	0.0072	0.0115
	Fecal	24	9999	3.351	0.04	0.0473
Jejunum	Ileum	22	9999	2.943	0.0617	0.0702
	Cecum	24	9999	32.093	0.0001	0.0003
	Colon	24	9999	15.379	0.0001	0.0003
	Fecal	24	9999	8.971	0.0017	0.0032
Ileum	Cecum	22	9999	36.288	0.0001	0.0003
	Colon	22	9999	21.775	0.0001	0.0003
	Fecal	22	9999	13.828	0.0002	0.0005
Cecum	Colon	24	9999	3.372	0.0471	0.0550
	Fecal	24	9999	4.256961	0.0330	0.0411
Colon	Fecal	24	9999	0.695824	0.4275	0.4275

Red highlights significance FDR-adjusted *q*-value < 0.05

Table S4C. Pairwise PERMANOVA test comparing weighted-UniFrac distances between segments of standard diet.

Group 1	Group 2	Sample size	Permutations	pseudo-F	p-value	q-value
Stomach	Duodenum	23	9999	1.7552	0.1684	0.1782
	Jejunum	24	9999	1.4433	0.2185	0.2285
	Ileum	23	9999	4.311	0.019	0.0251
	Cecum	24	9999	2.783	0.0646	0.073
	Colon	24	9999	4.379	0.0201	0.026
	Fecal	24	9999	9.071	0.001	0.001
Duodenum	Jejunum	23	9999	0.950	0.3612	0.369
	Ileum	22	9999	4.709	0.0185	0.025
	Cecum	23	9999	5.500	0.0018	0.003
	Colon	23	9999	2.367	0.1028	0.111
	Fecal	23	9999	3.840	0.0298	0.038
Jejunum	Ileum	23	9999	1.258	0.2696	0.279
	Cecum	24	9999	6.284	0.0038	0.007
	Colon	24	9999	5.879	0.0077	0.012
	Fecal	24	9999	8.637	0.0024	0.004
Ileum	Cecum	23	9999	15.158	0.0001	0.0003
	Colon	23	9999	14.750	0.0005	0.001
	Fecal	23	9999	20.198	0.0001	0.0003
Cecum	Colon	24	9999	5.394	0.0079	0.0122
	Fecal	24	9999	12.125	0.0001	0.0003
Colon	Fecal	24	9999	1.908	0.1302	0.139

Red highlights significance FDR-adjusted *q*-value < 0.05

Table S4D. Wilcoxon rank sum test of E.C. numbers between small and large intestines.

#OTU ID	E.C. description	Small intestine (n=68): mean rel. freq. (%)	Small intestine: std. dev. (%)	Large intestine (n=48): mean rel. freq. (%)	Large intestine: std. dev. (%)	q-values	Difference between means
small intestine							
EC:2.1.1.172	16S rRNA (guanine(1207)-N(2))-methyltransferase	0.119	0.045	0.032	0.024	2.80E-15	-0.086087074
EC:2.7.1.69	Protein-N(pi)-phosphohistidine--sugar phosphotransferase	1.198	0.402	0.420	0.226	1.70E-15	-0.778239965
EC:2.7.2.1	Acetate kinase	0.295	0.051	0.195	0.028	1.30E-15	-0.09946786
EC:2.7.4.9	dTMP kinase	0.142	0.041	0.069	0.023	3.80E-14	-0.07365658
EC:2.3.1.157	Glucosamine-1-phosphate N-acetyltransferase	0.142	0.041	0.061	0.028	1.10E-14	-0.08091178
EC:2.7.1.40	Pyruvate kinase	0.263	0.049	0.194	0.033	5.60E-11	-0.068373731
EC:3.1.26.8	Ribonuclease M5	0.131	0.043	0.055	0.028	3.80E-14	-0.076144848
EC:3.1.3.41	4-nitrophenylphosphatase	0.117	0.045	0.031	0.024	1.70E-15	-0.08637269
EC:3.2.1.86	6-phospho-beta-glucosidase	0.718	0.229	0.292	0.143	5.10E-14	-0.425569963
large intestine							
EC:1.1.1.29	Glycerate dehydrogenase	0.046	0.032	0.117	0.019	<2e-16	0.070757561
EC:1.1.1.69	Gluconate 5-dehydrogenase	0.032	0.032	0.081	0.037	1.40E-10	0.048615846
EC:1.4.1.16	Diaminopimelate dehydrogenase	0.036	0.035	0.096	0.018	2.20E-13	0.059361116
EC:2.1.1.132	Precorrin-6B C(5,15)-methyltransferase (decarboxylating)	0.019	0.027	0.074	0.030	2.50E-14	0.054808625
EC:4.1.1.3	Oxaloacetate decarboxylase	0.061	0.068	0.225	0.046	2.80E-16	0.163974128
EC:4.2.1.7	Altronate dehydratase	0.028	0.027	0.065	0.020	1.60E-10	0.036660183
EC:4.2.3.3	Methylglyoxal synthase	0.068	0.048	0.152	0.030	7.10E-14	0.08391944

Red highlights significance FDR-adjusted *q*-value < 0.05
