# Supplementary material for: Longitudinal Changes in Diet Cause Repeatable and Largely Reversible Shifts in Gut Microbial Communities of Laboratory Mice and Are Observed across Segments of the Entire Intestinal Tract
Source: Int J Mol Sci. 2021 Jun 1;22(11):5981. doi: 10.3390/ijms22115981 (PMC8198505; doi:10.3390/ijms22115981)
Supplement: Supplementary file 1 [file ijms-22-05981-s001.zip › Table S5.pdf]

Table S5A. ANCOM analysis of differentially abundant ASVs within segments of treatment and control group.

ASV ID	BLASTn Identity	clr*	W
**Stomach**			
ASV-834	Lactococcus taiwanensis strain 0905C15	8.15	435
ASV-654	Clostridium saudiense strain JCC	7.55	435
ASV-578	Clostridium saudiense strain JCC	6.51	432
ASV-479	Clostridium saudiense strain JCC	5.83	427
ASV-756	Clostridium saudiense strain JCC	5.43	421
ASV-540	Turicibacter sanguinis strain MOL361	5.37	391
ASV-603	[Clostridium] scindens strain ATCC 35704	5.05	425
ASV-542	[Eubacterium] rectale ATCC 33656	4.94	424
ASV-678	[Clostridium] scindens strain ATCC 35704	4.57	375
ASV-815	[Clostridium] asparagiforme strain N6	4.19	381
ASV-635	Anaerotignum lactatifermentans strain G17	3.64	371
ASV-501	[Clostridium] scindens strain ATCC 35704	3.63	381
ASV-564	Kineothrix alysoides strain KNHs209	3.58	380
ASV-862	Acetatifactor muris strain CT-m2	3.45	357
ASV-546	[Clostridium] scindens strain ATCC 35704	3.45	372
ASV-628	Anaerostipes caccae strain L1-92	3.40	366
ASV-791	Neglecta timonensis strain SN17	3.24	368
ASV-651	[Clostridium] scindens strain ATCC 35704	3.01	353
ASV-792	Bacteroides xylanolyticus strain X5-1	-3.32	353
ASV-895	Eisenbergiella tayi strain B086562	-3.39	358
ASV-708	Acetatifactor muris strain CT-m2	-3.46	352
ASV-574	Blautia faecicola strain KGMB01111	-3.75	380
ASV-644	Lachnoclostridium pacaense strain Marseille-P3100	-3.92	377
ASV-517	[Clostridium] scindens strain ATCC 35704	-4.38	380
ASV-746	Duncaniella dubosii strain H5	-4.54	397
ASV-733	Kineothrix alysoides strain KNHs209	-4.90	427
ASV-649	[Clostridium] asparagiforme strain N6	-4.95	407
ASV-526	Alistipes onderdonkii strain JCM 16771	-5.57	429
Duodenum			
ASV-834	Lactococcus taiwanensis strain 0905C15	7.48	365
ASV-654	Clostridium saudiense strain JCC	6.58	365
ASV-540	Turicibacter sanguinis strain MOL361	5.00	348
ASV-733	Kineothrix alysoides strain KNHs209	-4.70	346
ASV-517	[Clostridium] scindens strain ATCC 35704	-4.75	345
ASV-746	Duncaniella dubosii strain H5	-5.48	359
Jejunum			
ASV-834	Lactococcus taiwanensis strain 0905C15	8.84978	328
ASV-654	Clostridium saudiense strain JCC	6.42407	327
ASV-540	Turicibacter sanguinis strain MOL361	5.77653	321
ASV-578	Clostridium saudiense strain JCC	5.19645	327
ASV-479	Clostridium saudiense strain JCC	3.57336	291
ASV-756	Clostridium saudiense strain JCC	3.39088	290
ASV-496	Asaccharobacter celatus strain JCM 14811	3.38223	298
ASV-603	[Clostridium] scindens strain ATCC 35704	3.03965	308
ASV-815	[Clostridium] asparagiforme strain N6	2.84924	306
ASV-678	[Clostridium] scindens strain ATCC 35704	2.32251	292
ASV-791	Neglecta timonensis strain SN17	2.01916	280
ASV-651	[Clostridium] scindens strain ATCC 35704	2.01016	271
ASV-554	Blautia faecis strain M25	-2.4673	267
ASV-521	Marvinbryantia formatexigens strain I-52	-3.0688	279
ASV-575	Muricomes intestini strain 2PG-424-CC-1	-3.0857	284
ASV-798	Murimonas intestini strain SRB-530-5-H	-3.0969	289
ASV-695	Ruminococcus lactaris ATCC 29176	-3.2879	288
ASV-588	Paraeggerthella hongkongensis JCM 14552 strain HKU10	-3.5708	301
ASV-733	Kineothrix alysoides strain KNHs209	-3.5758	299
ASV-574	Blautia faecicola strain KGMB01111	-3.726	301
ASV-877	Murimonas intestini strain SRB-530-5-H	-3.8224	303
ASV-517	[Clostridium] scindens strain ATCC 35704	-4.9538	317
ASV-746	Duncaniella dubosii strain H5	-5.8069	324
Ileum			
ASV-834	Lactococcus taiwanensis strain 0905C15	8.7069	193
ASV-654	Clostridium saudiense strain JCC	6.96201	192
ASV-540	Turicibacter sanguinis strain MOL361	5.81858	189
ASV-526	Clostridium saudiense strain JCC	4.60089	178
ASV-746	Duncaniella dubosii strain H5	-5.9904	190
Cecum			
ASV-834	Lactococcus taiwanensis strain 0905C15	7.43633	460
ASV-678	[Clostridium] scindens strain ATCC 35704	6.98061	428
ASV-542	[Eubacterium] rectale ATCC 33656	6.38162	440
ASV-815	[Clostridium] asparagiforme strain N6	6.325	418
ASV-603	[Clostridium] scindens strain ATCC 35704	6.28349	428
ASV-654	Clostridium saudiense strain JCC	6.12195	436
ASV-564	Kineothrix alysoides strain KNHs209	5.79591	417
ASV-558	[Clostridium] scindens strain ATCC 35704	5.67119	438
ASV-862	Acetatifactor muris strain CT-m2	5.65718	414
ASV-626	Pseudobutyrivibrio ruminis strain DSM 9787	5.58964	413
ASV-546	[Clostridium] scindens strain ATCC 35704	5.46683	431
ASV-706	[Clostridium] methylpentosum strain R2	5.35642	416
ASV-653	Faecalimonas umbilicata strain EGH7	5.30622	362
ASV-791	Neglecta timonensis strain SN17	5.285	419
ASV-577	[Clostridium] scindens strain ATCC 35704	4.97934	415
ASV-595	[Clostridium] scindens strain ATCC 35704	4.9381	415
ASV-651	[Clostridium] scindens strain ATCC 35704	4.92577	414
ASV-515	[Eubacterium] rectale ATCC 33656	4.90725	415
ASV-540	Turicibacter sanguinis strain MOL361	4.89784	371
ASV-562	Roseburia intestinalis L1-82	4.88669	367
ASV-518	Phocea massiliensis strain Marseille-P2769	4.7916	401
ASV-854	Acetatifactor muris strain CT-m2	4.79106	404
ASV-681	Murimonas intestini strain SRB-530-5-H	4.78948	411
ASV-526	Clostridium saudiense strain JCC	4.73888	388
ASV-636	[Clostridium] asparagiforme strain N6	4.72845	391
ASV-774	[Eubacterium] rectale ATCC 33656	4.59478	407
ASV-279	Acetatifactor muris strain CT-m2	4.42648	383
ASV-522	Fusicatenibacter saccharivorans strain HT03-11	4.36441	370
ASV-612	Fusicatenibacter saccharivorans strain HT03-11	4.34419	368
ASV-635	Anaerotignum lactatifermentans strain G17	4.30486	377
ASV-500	Extibacter muris strain 40cc-B-5824-ARE	4.26962	368
ASV-841	Anaerotruncus rubiinfantis strain MT15	4.23883	403
ASV-722	Phocea massiliensis strain Marseille-P2769	4.14799	375
ASV-345	[Clostridium] scindens strain ATCC 35704	4.1288	353
ASV-822	[Clostridium] amygdalinum strain BR-10	3.92265	356
ASV-628	Anaerostipes caccae strain L1-92	3.75191	379
ASV-823	[Clostridium] algidixylanolyticum strain SPL73	3.46523	377
ASV-642	[Clostridium] leptum strain DSM 753	3.39343	364
ASV-354	Anaerotruncus colihominis strain WAL 14565	2.94023	347
ASV-578	Harryflintia acetispora strain V20-281a	2.79771	347
ASV-798	Murimonas intestini strain SRB-530-5-H	-3.8339	358
ASV-691	Intestinibacillus massiliensis strain Marseille-P3216	-3.9647	366
ASV-753	Roseburia hominis A2-183	-3.9795	370
ASV-731	[Clostridium] scindens strain ATCC 35704	-4.0234	361
ASV-344	Anaerotaenia torta strain FH052	-4.1527	384
ASV-834	Muricomes intestini strain 2PG-424-CC-1	-4.2146	391
ASV-792	Bacteroides xylanolyticus strain X5-1	-4.237	385
ASV-877	Murimonas intestini strain SRB-530-5-H	-4.2427	387
ASV-809	Anaeroplasma bactoclasticum strain JR	-4.253	395
ASV-574	Blautia faecicola strain KGMB01111	-4.4011	388
ASV-539	[Clostridium] aldenense strain RMA 9741	-4.4189	392
ASV-874	Roseburia intestinalis L1-82	-4.4426	397
ASV-895	Eisenbergiella tayi strain B086562	-5.0762	427
ASV-644	Lachnoclostridium pacaense strain Marseille-P3100	-5.2816	434
ASV-575	Alistipes onderdonkii strain JCM 16771	-5.8671	455
ASV-733	Kineothrix alysoides strain KNHs209	-6.6002	462
ASV-649	[Clostridium] asparagiforme strain N6	-7.0004	462
Colon			
ASV-834	Lactococcus taiwanensis strain 0905C15	7.8446	463
ASV-678	[Clostridium] scindens strain ATCC 35704	6.71699	446
ASV-654	Clostridium saudiense strain JCC	6.41597	456
ASV-603	[Clostridium] scindens strain ATCC 35704	6.18609	452
ASV-542	[Eubacterium] rectale ATCC 33656	6.05788	452
ASV-815	[Clostridium] asparagiforme strain N6	6.04324	430
ASV-578	Clostridium saudiense strain JCC	5.43171	443
ASV-564	Kineothrix alysoides strain KNHs209	5.18454	410
ASV-546	[Clostridium] scindens strain ATCC 35704	4.99265	436
ASV-540	Turicibacter sanguinis strain MOL361	4.96008	383
ASV-862	Acetatifactor muris strain CT-m2	4.88418	399
ASV-501	[Clostridium] scindens strain ATCC 35704	4.8669	422
ASV-635	Anaerotignum lactatifermentans strain G17	4.78195	404
ASV-653	Faecalimonas umbilicata strain EGH7	4.73388	362
ASV-626	Pseudobutyrivibrio ruminis strain DSM 9787	4.7066	398
ASV-562	Roseburia intestinalis L1-82	4.57395	374
ASV-791	Neglecta timonensis strain SN17	4.4431	407
ASV-706	[Clostridium] methylpentosum strain R2	4.37904	403
ASV-636	[Clostridium] asparagiforme strain N6	4.37358	390
ASV-651	[Clostridium] scindens strain ATCC 35704	4.36887	412
ASV-756	Clostridium saudiense strain JCC	4.33975	381
ASV-515	[Eubacterium] rectale ATCC 33656	4.32743	407
ASV-577	[Clostridium] scindens strain ATCC 35704	4.29865	408
ASV-479	Clostridium saudiense strain JCC	4.17662	369
ASV-595	[Clostridium] scindens strain ATCC 35704	4.15084	393
ASV-518	Phocea massiliensis strain Marseille-P2769	4.09091	381
ASV-279	Acetatifactor muris strain CT-m2	4.07757	391
ASV-522	Fusicatenibacter saccharivorans strain HT03-11	4.02568	390
ASV-612	Fusicatenibacter saccharivorans strain HT03-11	3.91294	396
ASV-681	Murimonas intestini strain SRB-530-5-H	3.85038	398
ASV-628	Anaerostipes caccae strain L1-92	3.768	381
ASV-854	Acetatifactor muris strain CT-m2	3.65927	371
ASV-722	Phocea massiliensis strain Marseille-P2769	3.5043	366
ASV-822	[Clostridium] amygdalinum strain BR-10	3.43326	353
ASV-599	Oscillibacter ruminantium GH1	3.06444	358
ASV-830	Faecalimonas umbilicata strain EGH7	3.02352	352
ASV-691	Intestinibacillus massiliensis strain Marseille-P3216	-3.3242	359
ASV-575	Muricomes intestini strain 2PG-424-CC-1	-3.6988	371
ASV-539	[Clostridium] aldenense strain RMA 9741	-3.7447	369
ASV-798	Murimonas intestini strain SRB-530-5-H	-3.8241	378
ASV-809	Anaeroplasma bactoclasticum strain JR	-3.8345	378
ASV-746	Duncaniella dubosii strain H5	-3.8567	364
ASV-558	Anaerotaenia torta strain FH052	-4.1676	401
ASV-895	Eisenbergiella tayi strain B086562	-4.2435	396
ASV-877	Murimonas intestini strain SRB-530-5-H	-4.3222	394
ASV-574	Blautia faecicola strain KGMB01111	-4.694	427
ASV-644	Lachnoclostridium pacaense strain Marseille-P3100	-4.9262	431
ASV-733	Kineothrix alysoides strain KNHs209	-6.2221	462
ASV-649	[Clostridium] asparagiforme strain N6	-6.2273	459
ASV-526	Alistipes onderdonkii strain JCM 16771	-6.4319	462

*negative CLR indicates differential abundance for control group.

Table S5B. ANCOM analysis of differentially abundant Metacyc pathways within segments of treatment and control group.

Metacyc ID	Metacyc pathway	clr*	W
**Stomach**			
PWY-6876	isopropanol biosynthesis	7.82	285
PWY-4984	urea cycle	7.33	285
PWY-7377	cob(II)yrinate a,c-diamide biosynthesis I (early cobalt insertion)	6.95	285
P441-PWY	superpathway of N-acetylneuraminate degradation	-4.49	275
PWY-7371	1,4-dihydroxy-6-naphthoate biosynthesis II	-5.49	284
PWY-6263	superpathway of menaquinol-8 biosynthesis II	-5.90	286
Duodenum			
PWY-4984	urea cycle	6.35	292
PWY-6876	isopropanol biosynthesis	6.82	292
PWY-7377	cob(II)yrinate a,c-diamide biosynthesis I (early cobalt insertion)	6.89	292
Jejunum			
PWY-6876	isopropanol biosynthesis	6.89	287
PWY-4984	urea cycle	5.81	286
PWY-7377	cob(II)yrinate a,c-diamide biosynthesis I (early cobalt insertion)	6.42	286
PWY-6263	superpathway of menaquinol-8 biosynthesis II	-3.07	273
Ileum			
PWY-6478	GDP-D-glycero-α-D-manno-heptose biosynthesis	4.05	249
PWY-6353	purine nucleotides degradation II (aerobic)	4.45	232
SALVADEHYF	adenosine nucleotides degradation II	4.83	252
PWY-6588	pyruvate fermentation to acetone	5.01	251
PWY-4984	urea cycle	5.74	251
PWY-7377	cob(II)yrinate a,c-diamide biosynthesis I (early cobalt insertion)	7.14	256
PWY-6876	isopropanol biosynthesis	7.57	256
Cecum			
PWY-5677	succinate fermentation to butanoate	5.09	257
PWY-5022	4-aminobutanoate degradation V	4.85	257
PWY-6263	superpathway of menaquinol-8 biosynthesis II	4.78	249
PWY-7371	1,4-dihydroxy-6-naphthoate biosynthesis II	4.65	249
P162-PWY	L-glutamate degradation V (via hydroxyglutarate)	4.62	257
PWY-7237	myo-, chiro- and scillo-inositol degradation	2.42	245
PWY-7377	cob(II)yrinate a,c-diamide biosynthesis I (early cobalt insertion)	-4.48	237
PWY-6470	peptidoglycan biosynthesis V (β-lactam resistance)	-4.98	243
PWY-5265	peptidoglycan biosynthesis II (staphylococci)	-5.19	244
PWY-6876	isopropanol biosynthesis	-6.57	259
PWY-4984	urea cycle	-6.58	259
Colon			
PWY-6876	isopropanol biosynthesis	6.81	262
PWY-4984	urea cycle	6.60	262
PWY-7377	cob(II)yrinate a,c-diamide biosynthesis I (early cobalt insertion)	5.26	251
PWY-6470	peptidoglycan biosynthesis V (β-lactam resistance)	3.28	237
PWY-5265	peptidoglycan biosynthesis II (staphylococci)	2.99	236
PWY-7237	myo-, chiro- and scillo-inositol degradation	-2.72	242
PWY-7431	aromatic biogenic amine degradation (bacteria)	-3.18	245
P162-PWY	L-glutamate degradation V (via hydroxyglutarate)	-3.21	242
PWY-5677	succinate fermentation to butanoate	-4.54	255
PWY-7371	1,4-dihydroxy-6-naphthoate biosynthesis II	-5.15	253
PWY-6263	superpathway of menaquinol-8 biosynthesis II	-5.39	252

*negative CLR indicates differential abundance for control group.
