# Supplementary material for: Longitudinal Changes in Diet Cause Repeatable and Largely Reversible Shifts in Gut Microbial Communities of Laboratory Mice and Are Observed across Segments of the Entire Intestinal Tract
Source: Int J Mol Sci. 2021 Jun 1;22(11):5981. doi: 10.3390/ijms22115981 (PMC8198505; doi:10.3390/ijms22115981)
Supplement: Supplementary file 1 [file ijms-22-05981-s001.zip › Table S1.pdf]

Table S1A. Weight and length of mice at the end of 84 days of the experiment.

Mouse	Diet	Host weight (g)	Length of segments (cm)				Total length (cm)
			Stomach	Small Intestine (Duodenum+Jejunum+Ileum)	Cecum	Colon	
M1	Standard	31.5	1.5	32.0	4.0	6.5	44.0
M2	Standard	28.5	1.5	34.5	3.5	6.0	45.5
M3	Standard	32.0	1.5	35.0	3.5	7.0	47.0
M4	Standard	34.1	1.5	34.0	3.5	6.5	45.5
M5	Standard	33.3	1.5	36.0	3.5	8.0	49.0
M6	Standard	34.5	2.0	37.5	3.5	7.5	50.5
M7	Standard	33.4	1.5	35.0	3.5	7.5	47.5
M8	Standard	31.3	1.5	36.5	3.5	7.5	49.0
M9	Standard	34.5	1.5	37.0	3.0	6.5	48.0
M10	Standard	33.0	1.5	37.5	3.0	6.5	48.5
M11	Standard	28.8	2.0	33.0	3.0	6.0	44.0
M12	Standard	34.1	1.5	33.0	3.0	6.5	44.0
M13	Western	41.1	1.0	29.5	2.0	5.0	37.5
M14	Western	39.6	1.5	32.0	1.5	4.0	39.0
M15	Western	40.8	1.5	31.0	1.5	5.0	39.0
M16	Western	39.8	1.0	29.5	1.5	5.0	37.0
M17	Western	32.2	1.0	24.5	1.5	5.0	32.0
M18	Western	45.4	1.5	29.5	1.5	6.0	38.5
M19	Western	36.2	1.0	27.0	1.5	5.0	34.5
M20	Western	38.7	1.0	29.5	1.5	4.5	36.5
M21	Western	38.7	1.0	28.5	1.0	5.0	35.5
M22	Western	36.7	1.5	30.0	2.0	5.5	39.0
M23	Western	40.3	1.5	28.5	2.0	4.5	36.5
M24	Western	37.9	1.5	29.0	1.5	5.0	37.0

Table S1B. Wilcoxon rank sum test on segment length.

Segment, Diet	Stomach, Standard	Stomach, Western	Small Intestine, Standard	Small Intestine, Western	Cecum, Standard	Cecum, Western	Colon, Standard	Colon, Western
Stomach, Standard	-	-	-	-	-	-	-	-
Stomach, Western	0.0042	-	-	-	-	-	-	-
Small Intestine, Standard	3.80E-05	3.80E-05	-	-	-	-	-	-
Small Intestine, Western	3.80E-05	3.80E-05	4.40E-05	-	-	-	-	-
Cecum, Standard	3.80E-05	3.80E-05	3.80E-05	3.80E-05	-	-	-	-
Cecum, Western	0.9695	0.0121	3.80E-05	3.80E-05	3.80E-05	-	-	-
Colon, Standard	3.80E-05	3.80E-05	3.90E-05	3.90E-05	3.80E-05	3.80E-05	-	-
Colon, Western	3.80E-05	3.80E-05	3.80E-05	3.80E-05	3.80E-05	3.80E-05	3.90E-05	-

significance FDR-adjusted *P*-value < 0.05

Table S1C. Wilcoxon rank sum test on segment lengths normalized to animal weight.

Segment, Diet	Stomach, Standard	Stomach, Western	Small Intestine, Standard	Small Intestine, Western	Cecum, Standard	Cecum, Western	Colon, Standard	Colon, Western
Stomach,Standard	-	-	-	-	-	-	-	-
Stomach,Western	4.10E-05	-	-	-	-	-	-	-
Small Intestine,Standard	4.10E-05	4.10E-05	-	-	-	-	-	-
Small Intestine,Western	4.10E-05	4.10E-05	2.30E-06	-	-	-	-	-
Cecum,Standard	4.10E-05	4.10E-05	2.30E-06	2.30E-06	-	-	-	-
Cecum,Western	0.0303	0.0123	2.30E-06	2.30E-06	2.30E-06	-	-	-
Colon,Standard	4.10E-05	4.10E-05	4.10E-05	4.10E-05	4.10E-05	4.10E-05	-	-
Colon,Western	4.10E-05	4.10E-05	2.30E-06	2.30E-06	0.0015	2.30E-06	4.10E-05	-

significance FDR-adjusted *P*-value < 0.05
