# Supplementary figures and images for: Longitudinal Changes in Diet Cause Repeatable and Largely Reversible Shifts in Gut Microbial Communities of Laboratory Mice and Are Observed across Segments of the Entire Intestinal Tract
Source: Int J Mol Sci. 2021 Jun 1;22(11):5981. doi: 10.3390/ijms22115981 (PMC8198505; doi:10.3390/ijms22115981)

A

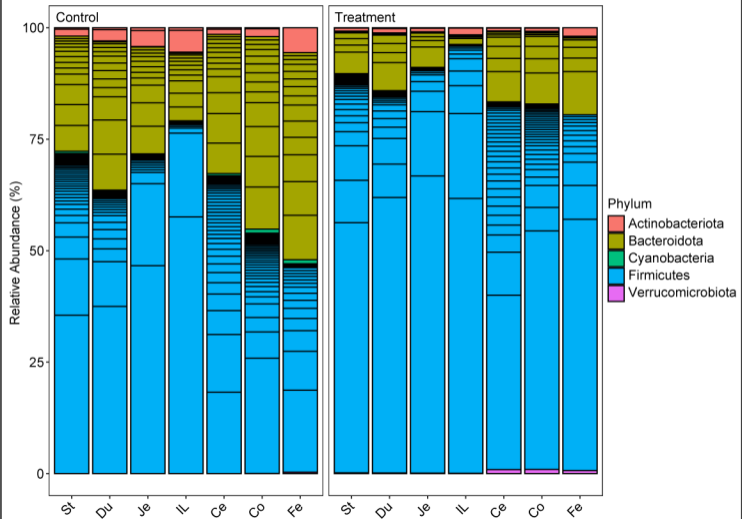

B

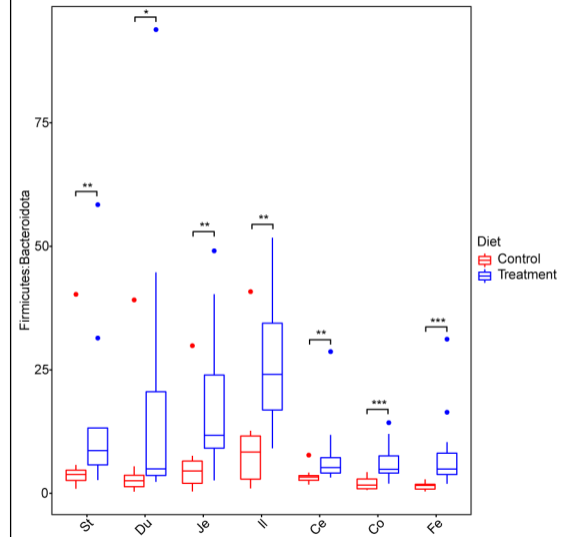

Supplement: Supplementary file 1 [file ijms-22-05981-s001.zip › Figure S1.pdf]

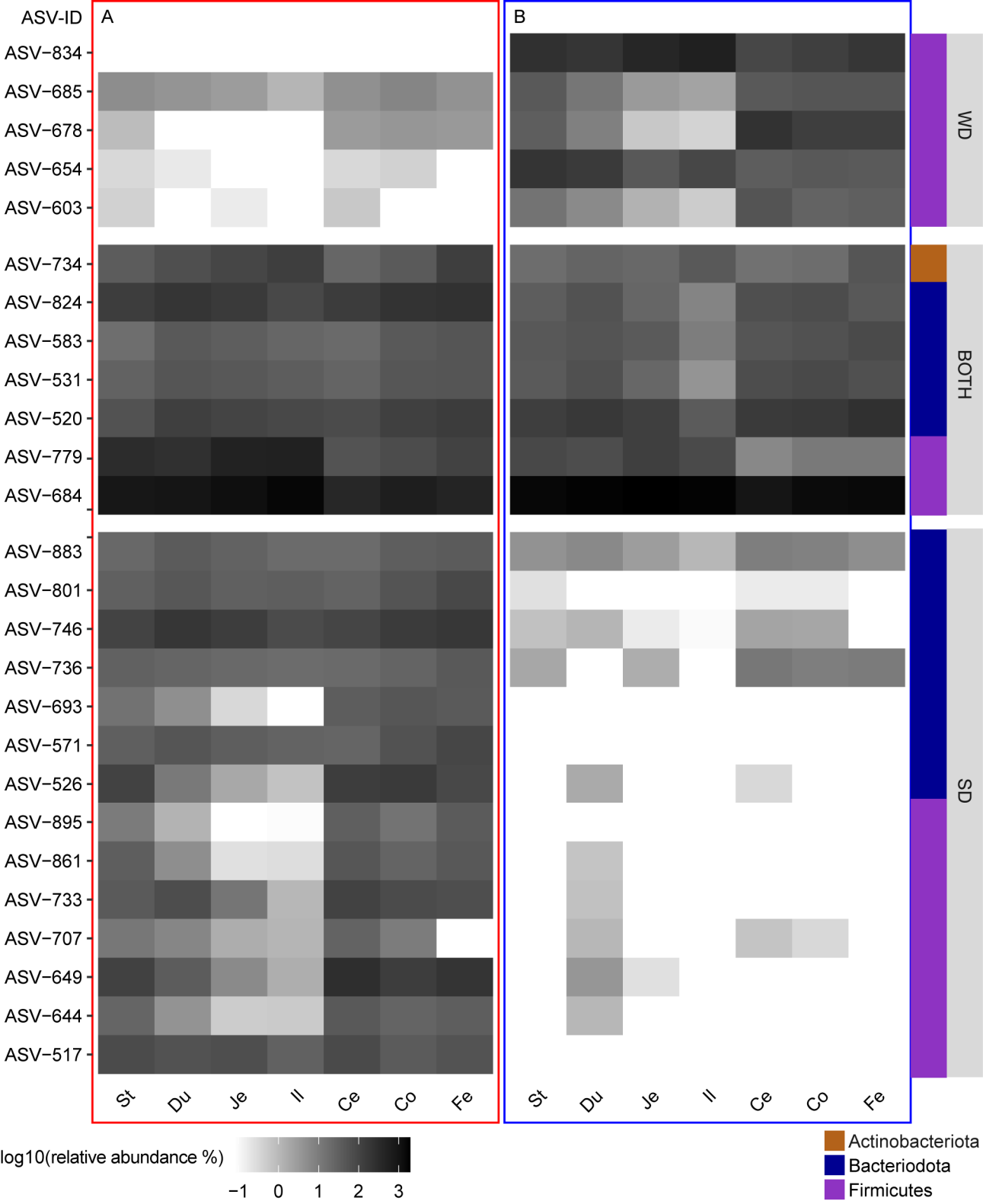

Supplement: Supplementary file 1 [file ijms-22-05981-s001.zip › Figure S2.pdf]

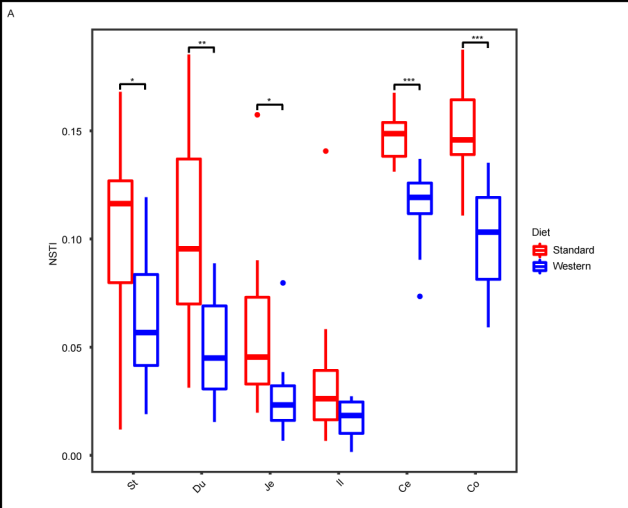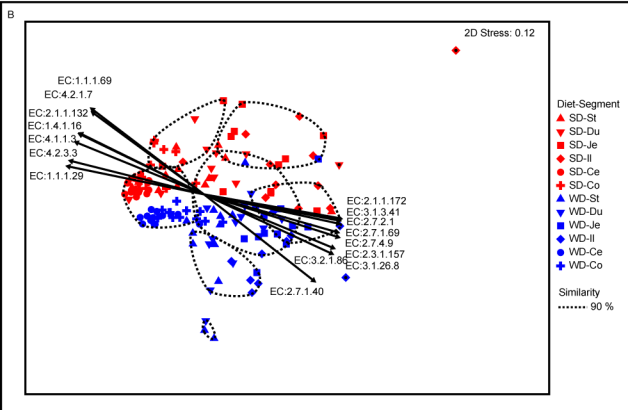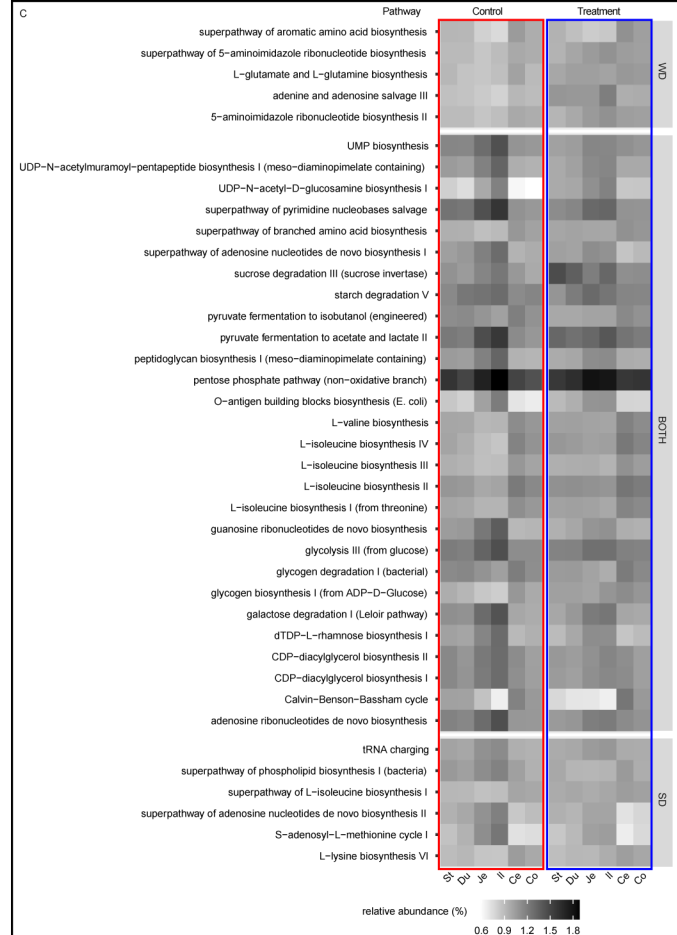

Supplement: Supplementary file 1 [file ijms-22-05981-s001.zip › Figure S3.pdf]
