# Supplementary material for: Longitudinal Changes in Diet Cause Repeatable and Largely Reversible Shifts in Gut Microbial Communities of Laboratory Mice and Are Observed across Segments of the Entire Intestinal Tract
Source: Int J Mol Sci. 2021 Jun 1;22(11):5981. doi: 10.3390/ijms22115981 (PMC8198505; doi:10.3390/ijms22115981)
Supplement: Supplementary file 1 [file Table S6.pdf › Table S6.pdf]

Table S6A. Mapping file showing metadata and number of quality filtered reads used for fecal sample analysis.

#SampleID	BarcodeSequence	Treatment or Control	Time (days)	Number of quality filtered reads
M01-3-Jul	TCAAGTCCGCAC	Control	27	7321
M05-3-Jul	TAGTTCGGTGAC	Treatmer	27	10129
M12-3-Jul	ACCTACTTGTCT	Treatmer	27	10467
M13-3-Jul	GGAGAACGACAC	Treatmer	27	7953
M16-3-Jul	TTAATGGATCGG	Control	27	8555
M20-3-Jul	ACTGTGACGTCC	Control	27	11323
M21-3-Jul	CCTACCATTGTT	Control	27	10616
M23-3-Jul	CATTTCGCACTT	Control	27	10095
M25-3-Jul	GTCCGTCCTGGT	Control	27	8151
M26-3-Jul	CTAGCGTGCGTT	Control	27	5539
M28-3-Jul	GTTCTAAGGTGA	Control	27	9993
M78-3-Jul	TGAGTCATTGAG	Treatmer	27	11315
M79-3-Jul	TCCACAGGGTTC	Treatmer	27	10269
M80-3-Jul	AGATCGTGCCTA	Treatmer	27	9980
M81-3-Jul	CCTCACTAGCGA	Treatmer	27	10890
M82-3-Jul	ACTAAAGCAAAC	Treatmer	27	10175
M83-3-Jul	CTTGCTCATAAT	Treatmer	27	9442
M85-3-Jul	GGTAGTTCATAG	Treatmer	27	11181
M86-3-Jul	AATTAATATGTA	Treatmer	27	8734
M89-3-Jul	CTAATCAGAGTG	Treatmer	27	10660
M91-3-Jul	TAGGAACTCACC	Control	27	7958
M92-3-Jul	ACGACAACGGGC	Control	27	7531
M93-3-Jul	CGATGAATATCG	Control	27	10739
M95-3-Jul	CTACTCCACGAG	Control	27	8518
M01-31-Jul	CAAACCTATGGC	Control	55	10037
M05-31-Jul	AGTGTTTCGGAC	Treatmer	55	9335
M12-31-Jul	GTCGCTTGCACA	Treatmer	55	7659
M13-31-Jul	TCTTGCGGAGTC	Treatmer	55	9053
M16-31-Jul	ATTTCCGCTAAT	Control	55	10767
M20-31-Jul	TCCGCCTAGTCG	Control	55	8649
M21-31-Jul	CTATCTCCTGTC	Control	55	9685
M23-31-Jul	GATATACCAGTG	Control	55	10072
M25-31-Jul	ATAGGAATAACC	Control	55	9194
M26-31-Jul	CACAACCACAAC	Control	55	36334
M28-31-Jul	TCGTTGGGACTA	Control	55	21043
M78-31-Jul	GCGCGGCGTTGC	Treatmer	55	10103
M79-31-Jul	AGACTTCTCAGG	Treatmer	55	10426
M80-31-Jul	AGTTCTCATTAA	Treatmer	55	10273
M81-31-Jul	GTGTCGAGGGCA	Treatmer	55	10888
M82-31-Jul	AGTGCAGGAGCC	Treatmer	55	10065
M83-31-Jul	GTTGCTGAGTCC	Treatmer	55	8960
M85-31-Jul	CGGAGAGACATG	Treatmer	55	11092
M86-31-Jul	GAGCCATCTGTA	Treatmer	55	8576
M89-31-Jul	TTCCACACGTGG	Treatmer	55	8605
M91-31-Jul	GTACTCGAACCA	Control	55	8834
M92-31-Jul	ACACCGCACAAT	Control	55	7296
M93-31-Jul	CAGCCCTACCCA	Control	55	9714
M95-31-Jul	AGAATCCACCAC	Control	55	9931
M01-28-Aug	GTTGGTTGGCAT	Control	83	7673
M05-28-Aug	ACATGGGCGGAA	Treatmer	83	8787
M12-28-Aug	TCATCCGTCGGC	Treatmer	83	15072
M13-28-Aug	GGCTTCGGAGCG	Treatmer	83	17467
M16-28-Aug	TGCGCGCCTTCC	Control	83	7257
M20-28-Aug	ATTTGGCTCTTA	Control	83	8496
M21-28-Aug	CGCGTCAAACTA	Control	83	7169
M23-28-Aug	ATGTTCCTCATC	Control	83	7295
M25-28-Aug	AACGGGCGACGT	Control	83	9284
M26-28-Aug	TACTGAGCCTCG	Control	83	8475
M28-28-Aug	GGTGCAGACAGA	Control	83	11215
M78-28-Aug	GCTAGTTATGGA	Treatmer	83	9439
M79-28-Aug	ACTAGTTGGACC	Treatmer	83	10419
M80-28-Aug	CGCAAGCCCGCG	Treatmer	83	5203
M81-28-Aug	AGAGCGGAACAA	Treatmer	83	9041
M82-28-Aug	CTGCGGATATAC	Treatmer	83	8786
M83-28-Aug	GTGAGGGCAAGT	Treatmer	83	10758
M85-28-Aug	ATATTGGCAGCC	Treatmer	83	10749
M86-28-Aug	CGGTCAATTGAC	Treatmer	83	8321
M89-28-Aug	TCCGCTGCTGAC	Treatmer	83	6752
M91-28-Aug	GCGCACACCTTC	Control	83	7011
M92-28-Aug	CACGAAAGCAGG	Control	83	10206
M93-28-Aug	CGTGGCTTTCCG	Control	83	7974
M95-28-Aug	TCCGTGGTATAG	Control	83	8372
			Mean count=	9935
			Standard deviation=	3889

Table S6B. Mapping file showing metadata used for segment analysis.

#SampleID	BarcodeSequence	Mouse	Gastroinstestinal segment	Diet*
P1.01-M1.St1	TACCGGCTTGCATGCGA	M1	stomach	SD
P1.24-M1.Du1	ATAGTTAGGGCTGAGTGG	M1	duodenum	SD
P1.36-M1.Je1	TGTTGCGTTTCTGT	M1	jejunum	SD
P1.48-M1.Je2	GCACTTCATTTCGAGTGG	M1	jejunum	SD
P1.60-M1.Je3	AGGTACGCAATTGT	M1	jejunum	SD
P1.72-M1.Il1	GAGACTATATGCCCTGGAG	M1	ileum	SD
P1.84-M1.Ce1	GAGGAGTAAAGCCGA	M1	cecum	SD
P2.01-M1.Ce2	GAGGAGTAAAGCCGA	M1	cecum	SD
P2.13-M1.Co1	CCATCACATAGG	M1	colon	SD
P2.25-M1.Co2	TGTTGCGTTTCTGT	M1	colon	SD
P1.10-M10.St1	CCTAAACTACGG	M10	stomach	SD
P1.21-M10.St2	ATCTAGTGGCAATGCGA	M10	stomach	SD
P1.33-M10.Du1	ACTTTAAGGGTGT	M10	duodenum	SD
P1.45-M10.Je1	CCAGGGACTTCTTGCGT	M10	jejunum	SD
P1.69-M10.Je3	CCAGGGACTTCTTGCGT	M10	jejunum	SD
P1.81-M10.Il1	AGGTACGCAATTGT	M10	ileum	SD
P1.93-M10.Ce1	GCACTTCATTTCGAGTGG	M10	cecum	SD
P2.10-M10.Ce2	GAGCAACATCCTT	M10	cecum	SD
P2.22-M10.Co1	CCATCACATAGG	M10	colon	SD
P2.34-M10.Co2	CGTAAGATGCCTATGA	M10	colon	SD
P1.22-M11.St2	CCAGGGACTTCTTGCGT	M11	stomach	SD
P1.46-M11.Je1	CACCTTACCTTAGAGTGG	M11	jejunum	SD
P1.58-M11.Je2	GAGCAACATCCTT	M11	jejunum	SD
P1.70-M11.Je3	ATAGTTAGGGCTGAGTGG	M11	jejunum	SD
P1.82-M11.Il1	ACAGCCACCCATCGA	M11	ileum	SD
P1.94-M11.Ce1	TTAACTGGAAGCCCTGTGG	M11	cecum	SD
P2.11-M11.Ce2	CCTAAACTACGG	M11	cecum	SD
P2.23-M11.Co1	GTGGTATGGGAGT	M11	colon	SD
P2.35-M11.Co2	TACCGGCTTGCATGCGA	M11	colon	SD
P1.12-M12.St1	TGTTGCGTTTCTGT	M12	stomach	SD
P1.23-M12.St2	CACCTTACCTTAGAGTGG	M12	stomach	SD
P1.35-M12.Du1	GAGCAACATCCTT	M12	duodenum	SD
P1.47-M12.Je1	ATAGTTAGGGCTGAGTGG	M12	jejunum	SD
P1.59-M12.Je2	TGTTGCGTTTCTGT	M12	jejunum	SD
P1.71-M12.Je3	CGCGGTTACTAACCTGGAG	M12	jejunum	SD
P1.83-M12.Il1	TGTCTCGCAAGCCGA	M12	ileum	SD
P1.95-M12.Ce1	CGCGGTTACTAACCTGGAG	M12	cecum	SD
P2.12-M12.Ce2	TGCAGATCCAAC	M12	cecum	SD
P2.24-M12.Co1	ACTTTAAGGGTGT	M12	colon	SD
P2.36-M12.Co2	ATCTAGTGGCAATGCGA	M12	colon	SD
P2.37-M13.St1	CCAGGGACTTCTTGCGT	M13	stomach	WD
P2.49-M13.St2	TGTTGCGTTTCTGT	M13	stomach	WD
P2.61-M13.Du1	TGTCTCGCAAGCCGA	M13	duodenum	WD
P2.84-M13.Je2	GAGGAGTAAAGCCGA	M13	jejunum	WD
P2.95-M13.Je3	AGGTACGCAATTGT	M13	jejunum	WD
P3.22-M13.Ce1	AGGTACGCAATTGT	M13	cecum	WD
P3.34-M13.Ce2	GTTACGTGGTTGATGA	M13	cecum	WD
P3.46-M13.Co1	TGTCTCGCAAGCCGA	M13	colon	WD
P3.58-M13.Co2	CGTAAGATGCCTATGA	M13	colon	WD
P2.38-M14.St1	CACCTTACCTTAGAGTGG	M14	stomach	WD
P2.50-M14.St2	ATGTCCGACCAAGT	M14	stomach	WD
P2.62-M14.Du1	GAGGAGTAAAGCCGA	M14	duodenum	WD
P2.73-M14.Je1	AGGTACGCAATTGT	M14	jejunum	WD
P2.85-M14.Je2	GTTACGTGGTTGATGA	M14	jejunum	WD
P3.01-M14.Je3	ACAGCCACCCATCGA	M14	jejunum	WD
P3.12-M14.Il1	GTTACGTGGTTGATGA	M14	ileum	WD
P3.23-M14.Ce1	ACAGCCACCCATCGA	M14	cecum	WD
P3.35-M14.Ce2	TACCGCCTCGGAATGA	M14	cecum	WD
P3.47-M14.Co1	GAGGAGTAAAGCCGA	M14	colon	WD
P3.59-M14.Co2	AGGTACGCAATTGT	M14	colon	WD
P2.39-M15.St1	ATAGTTAGGGCTGAGTGG	M15	stomach	WD
P2.51-M15.St2	AGGTACGCAATTGT	M15	stomach	WD
P2.63-M15.Du1	GTTACGTGGTTGATGA	M15	duodenum	WD
P2.74-M15.Je1	ACAGCCACCCATCGA	M15	jejunum	WD
P2.86-M15.Je2	TACCGCCTCGGAATGA	M15	jejunum	WD
P3.02-M15.Je3	TGTCTCGCAAGCCGA	M15	jejunum	WD
P3.13-M15.Il1	TACCGCCTCGGAATGA	M15	ileum	WD
P3.24-M15.Ce1	TGTCTCGCAAGCCGA	M15	cecum	WD
P3.36-M15.Ce2	CGTAAGATGCCTATGA	M15	cecum	WD
P3.48-M15.Co1	GTTACGTGGTTGATGA	M15	colon	WD
P3.60-M15.Co2	ACAGCCACCCATCGA	M15	colon	WD
P2.40-M16.St1	GCACTTCATTTCGAGTGG	M16	stomach	WD
P2.52-M16.St2	ACAGCCACCCATCGA	M16	stomach	WD
P2.64-M16.Du1	TACCGCCTCGGAATGA	M16	duodenum	WD
P2.75-M16.Je1	TGTCTCGCAAGCCGA	M16	jejunum	WD
P2.87-M16.Je2	CGTAAGATGCCTATGA	M16	jejunum	WD
P3.14-M16.Il1	CGTAAGATGCCTATGA	M16	ileum	WD
P3.25-M16.Ce1	GAGGAGTAAAGCCGA	M16	cecum	WD
P3.37-M16.Ce2	AGGTACGCAATTGT	M16	cecum	WD
P3.61-M16.Co2	TGTCTCGCAAGCCGA	M16	colon	WD
P2.41-M17.St1	TTAACTGGAAGCCCTGTGG	M17	stomach	WD
P2.65-M17.Du1	CGTAAGATGCCTATGA	M17	duodenum	WD
P3.04-M17.Je3	GAGGAGTAAAGCCGA	M17	jejunum	WD
P3.15-M17.Il1	AGGTACGCAATTGT	M17	ileum	WD
P3.38-M17.Ce2	ACAGCCACCCATCGA	M17	cecum	WD
P3.50-M17.Co1	TACCGCCTCGGAATGA	M17	colon	WD
P3.62-M17.Co2	GAGGAGTAAAGCCGA	M17	colon	WD
P2.42-M18.St1	CGCGGTTACTAACCTGGAG	M18	stomach	WD
P2.54-M18.St2	TGTCTCGCAAGCCGA	M18	stomach	WD
P2.66-M18.Du1	AGGTACGCAATTGT	M18	duodenum	WD
P2.77-M18.Je1	GAGGAGTAAAGCCGA	M18	jejunum	WD
P2.88-M18.Je2	AGGTACGCAATTGT	M18	jejunum	WD
P3.05-M18.Je3	GTTACGTGGTTGATGA	M18	jejunum	WD
P3.16-M18.Il1	ACAGCCACCCATCGA	M18	ileum	WD
P3.27-M18.Ce1	GTTACGTGGTTGATGA	M18	cecum	WD
P3.39-M18.Ce2	TGTCTCGCAAGCCGA	M18	cecum	WD
P3.51-M18.Co1	CGTAAGATGCCTATGA	M18	colon	WD
P3.63-M18.Co2	GTTACGTGGTTGATGA	M18	colon	WD
P2.43-M19.St1	GAGACTATATGCCCTGGAG	M19	stomach	WD
P2.55-M19.St2	GAGGAGTAAAGCCGA	M19	stomach	WD
P2.67-M19.Du1	ACAGCCACCCATCGA	M19	duodenum	WD
P2.78-M19.Je1	GTTACGTGGTTGATGA	M19	jejunum	WD
P2.89-M19.Je2	ACAGCCACCCATCGA	M19	jejunum	WD
P3.06-M19.Je3	TACCGCCTCGGAATGA	M19	jejunum	WD
P3.17-M19.Il1	TGTCTCGCAAGCCGA	M19	ileum	WD
P3.28-M19.Ce1	TACCGCCTCGGAATGA	M19	cecum	WD
P3.40-M19.Ce2	GAGGAGTAAAGCCGA	M19	cecum	WD
P3.52-M19.Co1	AGGTACGCAATTGT	M19	colon	WD
P3.64-M19.Co2	TACCGCCTCGGAATGA	M19	colon	WD
P1.02-M2.St1	ATCTAGTGGCAATGCGA	M2	stomach	SD
P1.13-M2.St2	ATGTCCGACCAAGT	M2	stomach	SD
P1.25-M2.Du1	GCACTTCATTTCGAGTGG	M2	duodenum	SD
P1.37-M2.Je1	ATGTCCGACCAAGT	M2	jejunum	SD
P1.49-M2.Je2	TTAACTGGAAGCCCTGTGG	M2	jejunum	SD
P1.61-M2.Je3	ACAGCCACCCATCGA	M2	jejunum	SD
P1.73-M2.Il1	CCTAAACTACGG	M2	ileum	SD
P1.85-M2.Ce1	GTTACGTGGTTGATGA	M2	cecum	SD
P2.02-M2.Ce2	GTTACGTGGTTGATGA	M2	cecum	SD
P2.14-M2.Co1	GTGGTATGGGAGT	M2	colon	SD
P2.26-M2.Co2	ATGTCCGACCAAGT	M2	colon	SD
P2.44-M20.St1	CCTAAACTACGG	M20	stomach	WD
P2.56-M20.St2	GTTACGTGGTTGATGA	M20	stomach	WD
P2.68-M20.Du1	TGTCTCGCAAGCCGA	M20	duodenum	WD
P2.79-M20.Je1	TACCGCCTCGGAATGA	M20	jejunum	WD
P2.90-M20.Je2	TGTCTCGCAAGCCGA	M20	jejunum	WD
P3.07-M20.Je3	CGTAAGATGCCTATGA	M20	jejunum	WD
P3.29-M20.Ce1	CGTAAGATGCCTATGA	M20	cecum	WD
P3.41-M20.Ce2	GTTACGTGGTTGATGA	M20	cecum	WD
P3.53-M20.Co1	ACAGCCACCCATCGA	M20	colon	WD
P2.45-M21.St1	TGCAGATCCAAC	M21	stomach	WD
P2.57-M21.St2	TACCGCCTCGGAATGA	M21	stomach	WD
P2.69-M21.Du1	GAGGAGTAAAGCCGA	M21	duodenum	WD
P2.80-M21.Je1	CGTAAGATGCCTATGA	M21	jejunum	WD
P2.91-M21.Je2	GAGGAGTAAAGCCGA	M21	jejunum	WD
P3.08-M21.Je3	AGGTACGCAATTGT	M21	jejunum	WD
P3.18-M21.Il1	GAGGAGTAAAGCCGA	M21	ileum	WD
P3.30-M21.Ce1	AGGTACGCAATTGT	M21	cecum	WD
P3.42-M21.Ce2	TACCGCCTCGGAATGA	M21	cecum	WD
P3.54-M21.Co1	TGTCTCGCAAGCCGA	M21	colon	WD
P3.65-M21.Co2	CGTAAGATGCCTATGA	M21	colon	WD
P2.46-M22.St1	CCATCACATAGG	M22	stomach	WD
P2.58-M22.St2	CGTAAGATGCCTATGA	M22	stomach	WD
P2.70-M22.Du1	GTTACGTGGTTGATGA	M22	duodenum	WD
P2.81-M22.Je1	AGGTACGCAATTGT	M22	jejunum	WD
P2.92-M22.Je2	GTTACGTGGTTGATGA	M22	jejunum	WD
P3.09-M22.Je3	ACAGCCACCCATCGA	M22	jejunum	WD
P3.19-M22.Il1	GTTACGTGGTTGATGA	M22	ileum	WD
P3.31-M22.Ce1	ACAGCCACCCATCGA	M22	cecum	WD
P3.43-M22.Ce2	CGTAAGATGCCTATGA	M22	cecum	WD
P3.55-M22.Co1	GAGGAGTAAAGCCGA	M22	colon	WD
P3.66-M22.Co2	AGGTACGCAATTGT	M22	colon	WD
P2.47-M23.St1	GTGGTATGGGAGT	M23	stomach	WD
P2.59-M23.St2	AGGTACGCAATTGT	M23	stomach	WD
P2.71-M23.Du1	TACCGCCTCGGAATGA	M23	duodenum	WD
P2.82-M23.Je1	ACAGCCACCCATCGA	M23	jejunum	WD
P2.93-M23.Je2	TACCGCCTCGGAATGA	M23	jejunum	WD
P3.10-M23.Je3	TGTCTCGCAAGCCGA	M23	jejunum	WD
P3.20-M23.Il1	TACCGCCTCGGAATGA	M23	ileum	WD
P3.32-M23.Ce1	TGTCTCGCAAGCCGA	M23	cecum	WD
P3.44-M23.Ce2	AGGTACGCAATTGT	M23	cecum	WD
P3.56-M23.Co1	GTTACGTGGTTGATGA	M23	colon	WD
P3.67-M23.Co2	ACAGCCACCCATCGA	M23	colon	WD
P2.48-M24.St1	GAGCAACATCCTT	M24	stomach	WD
P2.60-M24.St2	ACAGCCACCCATCGA	M24	stomach	WD
P2.72-M24.Du1	CGTAAGATGCCTATGA	M24	duodenum	WD
P2.83-M24.Je1	TGTCTCGCAAGCCGA	M24	jejunum	WD
P2.94-M24.Je2	CGTAAGATGCCTATGA	M24	jejunum	WD
P3.11-M24.Je3	GAGGAGTAAAGCCGA	M24	jejunum	WD
P3.21-M24.Il1	CGTAAGATGCCTATGA	M24	ileum	WD
P3.33-M24.Ce1	GAGGAGTAAAGCCGA	M24	cecum	WD
P3.45-M24.Ce2	ACAGCCACCCATCGA	M24	cecum	WD
P3.57-M24.Co1	TACCGCCTCGGAATGA	M24	colon	WD
P3.68-M24.Co2	TGTCTCGCAAGCCGA	M24	colon	WD
P1.03-M3.St1	CCAGGGACTTCTTGCGT	M3	stomach	SD
P1.14-M3.St2	AGGTACGCAATTGT	M3	stomach	SD
P1.26-M3.Du1	TTAACTGGAAGCCCTGTGG	M3	duodenum	SD
P1.38-M3.Je1	TGTCTCGCAAGCCGA	M3	jejunum	SD
P1.50-M3.Je2	CGCGGTTACTAACCTGGAG	M3	jejunum	SD
P1.62-M3.Je3	TGTCTCGCAAGCCGA	M3	jejunum	SD
P1.74-M3.Il1	TGCAGATCCAAC	M3	ileum	SD
P1.86-M3.Ce1	TACCGCCTCGGAATGA	M3	cecum	SD
P2.03-M3.Ce2	TACCGCCTCGGAATGA	M3	cecum	SD
P2.15-M3.Co1	ACTTTAAGGGTGT	M3	colon	SD
P2.27-M3.Co2	AGGTACGCAATTGT	M3	colon	SD
P1.04-M4.St1	CACCTTACCTTAGAGTGG	M4	stomach	SD
P1.15-M4.St2	TGTCTCGCAAGCCGA	M4	stomach	SD
P1.27-M4.Du1	CGCGGTTACTAACCTGGAG	M4	duodenum	SD
P1.39-M4.Je1	GAGGAGTAAAGCCGA	M4	jejunum	SD
P1.51-M4.Je2	GAGACTATATGCCCTGGAG	M4	jejunum	SD
P1.63-M4.Je3	GAGGAGTAAAGCCGA	M4	jejunum	SD
P1.75-M4.Il1	CCATCACATAGG	M4	ileum	SD
P1.87-M4.Ce1	CGTAAGATGCCTATGA	M4	cecum	SD
P2.04-M4.Ce2	CGTAAGATGCCTATGA	M4	cecum	SD
P2.16-M4.Co1	GAGCAACATCCTT	M4	colon	SD
P2.28-M4.Co2	ACAGCCACCCATCGA	M4	colon	SD
P1.05-M5.St1	ATAGTTAGGGCTGAGTGG	M5	stomach	SD
P1.16-M5.St2	GAGGAGTAAAGCCGA	M5	stomach	SD
P1.28-M5.Du1	GAGACTATATGCCCTGGAG	M5	duodenum	SD
P1.40-M5.Je1	GTTACGTGGTTGATGA	M5	jejunum	SD
P1.52-M5.Je2	CCTAAACTACGG	M5	jejunum	SD
P1.64-M5.Je3	GTTACGTGGTTGATGA	M5	jejunum	SD
P1.76-M5.Il1	GTGGTATGGGAGT	M5	ileum	SD
P1.88-M5.Ce1	TACCGGCTTGCATGCGA	M5	cecum	SD
P2.05-M5.Ce2	TGCAGATCCAAC	M5	cecum	SD
P2.17-M5.Co1	TGTTGCGTTTCTGT	M5	colon	SD
P2.29-M5.Co2	TGTCTCGCAAGCCGA	M5	colon	SD
P1.06-M6.St1	GCACTTCATTTCGAGTGG	M6	stomach	SD
P1.17-M6.St2	GTTACGTGGTTGATGA	M6	stomach	SD
[illegible]	[illegible]	[illegible]	[illegible]	[illegible]

*SD is standard diet and WD is western diet.

Table S6C. Quality filtered and merged (mean) samples of the same parts and mouse identity used in the study.

Sample-ID	Gastroinstestinal segment	Diet*	Number of quality filtered reads
St.M1	Stomach	SD	29659
St.M2	Stomach	SD	16862
St.M3	Stomach	SD	15302
St.M4	Stomach	SD	27515
St.M5	Stomach	SD	20986
St.M6	Stomach	SD	26346
St.M7	Stomach	SD	27127
St.M8	Stomach	SD	28624
St.M9	Stomach	SD	29455
St.M10	Stomach	SD	16706
St.M11	Stomach	SD	16178
St.M12	Stomach	SD	18603
St.M13	Stomach	WD	25493
St.M14	Stomach	WD	33963
St.M15	Stomach	WD	41922
St.M16	Stomach	WD	34803
St.M17	Stomach	WD	32447
St.M18	Stomach	WD	40884
St.M19	Stomach	WD	25620
St.M20	Stomach	WD	35671
St.M21	Stomach	WD	36874
St.M22	Stomach	WD	33705
St.M23	Stomach	WD	40168
St.M24	Stomach	WD	43318
Du.M1	Duodenum	SD	15162
Du.M2	Duodenum	SD	38403
Du.M3	Duodenum	SD	36155
Du.M4	Duodenum	SD	19457
Du.M5	Duodenum	SD	45652
Du.M6	Duodenum	SD	26230
Du.M7	Duodenum	SD	28669
Du.M8	Duodenum	SD	30425
Du.M9	Duodenum	SD	31489
Du.M10	Duodenum	SD	37036
Du.M12	Duodenum	SD	28734
Du.M13	Duodenum	WD	28097
Du.M14	Duodenum	WD	2945
Du.M15	Duodenum	WD	30975
Du.M16	Duodenum	WD	10538
Du.M17	Duodenum	WD	11854
Du.M18	Duodenum	WD	19452
Du.M19	Duodenum	WD	6886
Du.M20	Duodenum	WD	27418
Du.M21	Duodenum	WD	24570
Du.M22	Duodenum	WD	28656
Du.M23	Duodenum	WD	24129
Du.M24	Duodenum	WD	28633
Je.M1	Jejunum	SD	50920
Je.M2	Jejunum	SD	44434
Je.M3	Jejunum	SD	26047
Je.M4	Jejunum	SD	31963
Je.M5	Jejunum	SD	35135
Je.M6	Jejunum	SD	37458
Je.M7	Jejunum	SD	36437
Je.M8	Jejunum	SD	35338
Je.M9	Jejunum	SD	24353
Je.M10	Jejunum	SD	26213
Je.M11	Jejunum	SD	29889
Je.M12	Jejunum	SD	30463
Je.M13	Jejunum	WD	28207
Je.M14	Jejunum	WD	50205
Je.M15	Jejunum	WD	40149
Je.M16	Jejunum	WD	39926
Je.M17	Jejunum	WD	32772
Je.M18	Jejunum	WD	36662
Je.M19	Jejunum	WD	34858
Je.M20	Jejunum	WD	40340
Je.M21	Jejunum	WD	34037
Je.M22	Jejunum	WD	35584
Je.M23	Jejunum	WD	37621
Je.M24	Jejunum	WD	54234
Il.M1	Ileum	SD	55323
Il.M2	Ileum	SD	60593
Il.M3	Ileum	SD	44668
Il.M4	Ileum	SD	30031
Il.M5	Ileum	SD	49287
Il.M6	Ileum	SD	41858
Il.M7	Ileum	SD	47048
Il.M8	Ileum	SD	45349
Il.M10	Ileum	SD	26326
Il.M11	Ileum	SD	45521
Il.M12	Ileum	SD	31888
Il.M14	Ileum	WD	76976
Il.M15	Ileum	WD	32235
Il.M16	Ileum	WD	11104
Il.M17	Ileum	WD	29743
Il.M18	Ileum	WD	26838
Il.M19	Ileum	WD	33433
Il.M21	Ileum	WD	18064
Il.M22	Ileum	WD	24527
Il.M23	Ileum	WD	31795
Il.M24	Ileum	WD	42052
Ce.M1	Cecum	SD	57670
Ce.M2	Cecum	SD	38003
Ce.M3	Cecum	SD	36730
Ce.M4	Cecum	SD	40095
Ce.M5	Cecum	SD	32246
Ce.M6	Cecum	SD	36397
Ce.M7	Cecum	SD	35856
Ce.M8	Cecum	SD	26504
Ce.M9	Cecum	SD	37779
Ce.M10	Cecum	SD	22631
Ce.M11	Cecum	SD	39191
Ce.M12	Cecum	SD	37822
Ce.M13	Cecum	WD	34616
Ce.M14	Cecum	WD	39963
Ce.M15	Cecum	WD	43932
Ce.M16	Cecum	WD	34874
Ce.M17	Cecum	WD	21554
Ce.M18	Cecum	WD	27709
Ce.M19	Cecum	WD	35166
Ce.M20	Cecum	WD	31234
Ce.M21	Cecum	WD	36647
Ce.M22	Cecum	WD	30949
Ce.M23	Cecum	WD	37416
Ce.M24	Cecum	WD	31738
Co.M1	Colon	SD	34366
Co.M2	Colon	SD	35991
Co.M3	Colon	SD	35383
Co.M4	Colon	SD	38951
Co.M5	Colon	SD	32721
Co.M6	Colon	SD	41546
Co.M7	Colon	SD	33085
Co.M8	Colon	SD	36336
Co.M9	Colon	SD	25554
Co.M10	Colon	SD	35565
Co.M11	Colon	SD	29964
Co.M12	Colon	SD	35478
Co.M13	Colon	SD	46383
Co.M14	Colon	WD	45765
Co.M15	Colon	WD	51351
Co.M16	Colon	WD	41847
Co.M17	Colon	WD	24154
Co.M18	Colon	WD	39499
Co.M19	Colon	WD	39309
Co.M20	Colon	WD	38908
Co.M21	Colon	WD	41833
Co.M22	Colon	WD	43459
Co.M23	Colon	WD	44050
Co.M24	Colon	WD	33781
		Mean count=	33641
		Standard deviation=	10519

*SD is standard diet and WD is western diet.
